# Atomic‐Level CuO_x_‐CoO_x_‐Pd Interfacial Engineering Enables Hierarchical Synergy for High‐Efficiency ORR Pathways and Boosted Power Output in Alkaline Fuel Cells

**DOI:** 10.1002/advs.76006

**Published:** 2026-06-04

**Authors:** Yang‐Yang Hsu, Ching‐Hua Fan, Kuan‐Wen Wang, Kuang‐Kuo Wang, Nozomu Hiraoka, Hirofumi Ishii, Ting‐Shan Chan, Po‐Chun Chen, Tsan‐Yao Chen

**Affiliations:** ^1^ Department of Engineering and System Science National Tsing Hua University Hsinchu Taiwan; ^2^ Department of Materials and Mineral Resources Engineering National Taipei University of Technology Taipei Taiwan; ^3^ Institute of Materials Science and Engineering National Central University Taoyuan City Taiwan; ^4^ Department of Materials and Optoelectronic National Sun Yat‐sen University Kaohsiung Taiwan; ^5^ National Synchrotron Radiation Research Center Hsinchu Taiwan; ^6^ Institute of Analytical and Environmental Science National Tsing Hua University Hsinchu Taiwan; ^7^ Institute of Nuclear Engineering and Science National Tsing Hua University Hsinchu Taiwan; ^8^ Department of Chemical Engineering Chung‐Yuan Christian University Taoyuan City Taiwan

**Keywords:** catalysis, chemical engineering, materials science, oxygen reduction reaction, power density, xanes

## Abstract

The sluggish kinetics of the oxygen reduction reaction (ORR) and the high cost of Pt‐based catalysts remain major barriers for alkaline fuel cell (AFC) technologies. Here we report a Cu‐decorated CoO@Pd catalyst in which atomic‐to‐subnanometer CuO_x_ species reconstruct the Pd‐Co‐Cu heterointerface and enable a cooperative multi‐site ORR pathway. Structural and spectroscopic analyses reveal that low Cu loading produces atomically dispersed CuO_x_ motifs that enrich oxygen vacancies (O_V_)s, preserve metallic Pd, and stabilize oxygen‐deficient Co^3^^+^ centers. Operando PFY‐XANES/EXAFS further uncovers a synergistic mechanism in which O_V_s around Cu serve as rapid O_2_ activation sites, Pd mediates lateral *O^ads^ spillover, and O_V_s around Co act as the primary *O^ads^ reduction centers. This division of labor maximizes four‐electron ORR kinetics, yielding a on‐set potential of 0.935 V and a mass activity (MA) of ∼1.6 × 10^4^ mA mg_Cu_^−^^1^ without decay for 50k potential cycles, surpassing commercial Pt/C by over two orders of magnitude. When integrated into AFCs, the optimized catalyst (CPCu‐1) delivers a peak power density of ∼430 mW cm^−^^2^, approximately 60% higher than Pt/C, and exhibits a characteristic three‐stage durability governed by dynamic CuO_x_‐CoO_x_ interface reconstruction. These findings establish atomic CuO_x_ interface engineering strategy for designing high‐performance, noble‐metal‐efficient ORR catalysts.

## Graphical abstract

Atomically dispersed CuO_x_ (CPCu‐1) forms isolated Cu–O_v_ sites that enable defect‐mediated O_2_ activation and fast electron/proton transfer via interfacial synergy. In contrast, thick CuO_x_ clusters (CPCu‐10) promote a surface‐adsorption‐controlled pathway with intermediate accumulation, highlighting a mechanism switch from interfacial activation to adsorption‐dominated catalysis.

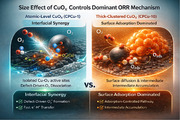

## 1 Introduction

In alkaline fuel cells, the oxygen reduction reaction (ORR) remains the central kinetic bottleneck governing energy‐conversion efficiency, cost structure, and long‐term operational stability. Although alkaline media reduce the dependence on platinum‐group metals, high‐power applications still demand cathode catalysts that combine rapid four‐electron kinetics with strong resistance to poisoning and robust durability. Because ORR involves multiple elementary steps including O_2_ activation, O─O bond scission, adsorption and transformation of *O^ads^/*OH^ads^ intermediates, and interfacial hydration. No single metallic site can simultaneously meet all energetic and geometric requirements [1, 2, 3, 4, 5, 6, 7, 8, 9]. Consequently, research efforts have increasingly shifted toward multi‐metallic and metal–oxide heterointerfaces, aiming to exploit interfacial band gradients, charge redistribution, defect chemistry, and ensemble cooperativity to surpass the intrinsic limitations of conventional Pd, single‐atom and dual‐atom catalysts [10, 11, 12, 13, 14, 15, 16]. Most existing designs (such as homogeneous multicomponent alloy nanoparticles (NPs) and core‐shell structures with uniform overlayers) feature highly ordered surface atomic arrangements in which all metal sites share similar electronic states and adsorption energies [17, 18]. This seemingly stable configuration introduces a critical drawback: reactants, oxygen intermediates, and even products compete for identical adsorption motifs (competitive chemisorption), lowering reaction selectivity and increasing side‐reaction probability [19]. For a multistep reaction such as ORR, this means O_2_ activation, O─O cleavage, *O^ads^ transformation, and OH* desorption must all occur on a homogenized surface. Without local variations in electronic states or coordination geometry, the catalyst cannot provide step‐specific adsorption energies (Sabatier tuning), leading to following reaction barriers including (1) inefficient O_2_ polarization and bond breaking; (2) over‐ or under‐stabilization of *O^ads^; (3) inadequate OH* desorption and subsequent poisoning and (4) intrinsic site‐level competition rather than a distributed reaction‐pathway architecture [20, 21, 22, 23, 24]. This fundamental weakness explains why modifying alloy compositions or shell thicknesses alone rarely yields truly high ORR kinetics. Furthermore, under realistic electrochemical operating potentials, most existing systems fail to sustain stable electronic coupling, persistent oxygen‐vacancy structures, or controlled intermediate flux. Traditional Pd‐based catalysts often bind *O^ads^ too strongly, lack sufficient interfacial charge modulation, and exhibit poor redistribution of oxygen species, resulting in localized poisoning and transport blockage. Transition‐metal oxides such as CoO_x_ possess high densities of O_V_s capable of promoting O_2_ activation, yet their insufficient electronic conductivity and suboptimal intermediate stability prevent them from independently supporting the full ORR sequence [2, 23].

Hybrid Pd‐MO_x_ structures partially alleviate these issues but frequently suffer from poorly defined interfaces, uncontrollable oxide‐layer thickness, and electronic shielding effects that partition reaction steps rather than forming an integrated multi‐site catalytic network. Likewise, single‐atom or ultrathin‐oxide decorations improve atomic utilization but remain limited by (i) the absence of ensemble sites capable of jointly handling O_2_ activation and *O^ads^ conversion; (ii) susceptibility to oxidation and structural rearrangement under working potentials; and (iii) inadequate electronic pathways (“electron highways”) and vacancy–metal coupling needed to sustain a continuous catalytic cycle. Recent studies suggest that precisely controlling O_V_s and interfacial electronic structure through defect engineering can reshape the O_2_ activation landscape and create hetero‐structured catalytic platforms featuring multi‐site task division and cross‐interface cooperation. However, there remains a lack of catalytic architectures that simultaneously integrate the (i) vacancy‐rich oxide domains, (ii) electronically flexible metal centers, and (iii) metallic pathways capable of regulating intermediate flux [25]. Such a system must also undergo spontaneous atomic‐scale interface reconstruction under operating potentials to enable synchronized progression of multiple reaction steps—forming a genuine multi‐site heterogeneous reaction mechanism [26].

The literature strongly supports this necessity. Among Ni‐based materials, only Ni(OH)_2_ provides sufficient lattice flexibility, surface polarity, and hydration capability to form strongly coupled interfaces with nearby Pd or Pt clusters, enabling effective *O^ads^ shuttling [27, 28]. Atomic‐scale oxide clusters such as Pt, IrO_x_, TiO_x_, and transition metal oxide have been shown to serve as highly active O_2_ activation and O─O bond‐cleavage sites, highlighting the critical role of nanoscale metal–oxide domains in the early ORR steps [29, 30, 31]. Notably, TiO_x_ clusters—with their multivalent states and hydrophilic surfaces—can simultaneously capture O_2_, adsorb water molecules, and facilitate *O^ads^ conversion, while neighboring Pd or Co(OH)_x_ motifs help absorb excess *O^ads^ through O_V_‐metal cooperativity [32]. However, the intrinsically strong *O^ads^ binding energy of TiO_x_ and the instability of its hydration chemistry introduce limitations in synthetic control and reaction stability. These collective insights underscore that a truly high‐performance ORR catalyst must achieve, at the interfacial level, cooperative O_2_ activation, O─O cleavage, *O^ads^ hydration, cross‐interface transfer, and electron rectification among Pd‐M‐O_x_ (M = Co or Cu). This requirement defines the conceptual entry point of the present study [33].

Here, we introduce an atomic‐scale CuO_x_ interface‐reconstruction strategy that allows precise structural control in aqueous media. By inducing CuO_x_ reconstruction on Pd NPs supported by CoO_x_, extremely low Cu loadings yield ultrathin, uniform, O_V_‐rich CuO_x_ layers featuring dispersed Cu─O─M (M = Pd/Co) linkages, electronically permeable interfaces, and dynamically adjustable defects. These features generate continuous Pd‐CoO_x_ electronic‐coupling pathways and cooperative multi‐site reaction domains. In contrast, excessive Cu loadings produce more insulating island‐type CuO_x_ domains, which partially block Pd‐CoO_x_ electronic transport and *O^ads^ mobility, illustrating the critical importance of atomic‐scale reconstruction. Through X‐ray absorption near‐edge structure (XANES)/Extended X‐ray absorption fine structure (EXAFS), X‐ray photoemission spectroscopy (XPS), and operando partial fluorescence yield (PFY)‐XANES/EXAFS analyses, we reveal that the optimally reconstructed CPCu‐1 interface operates through a tri‐metallic division‐of‐labor mechanism. Details for the aforementioned mechanisms will be systematically investigated in the latter sessions.

## 2 Experimental

### 2.1 Synthesis of CoO_x_ Supported Pd NPs with Atomic CuO_x_ Cluster Decoration

The CoO_x_ supported Pd NPs with atomic CuO_x_ cluster decoration (namely, CPCu‐1) was synthesized by chemical reduction reaction with sequence and reaction time control. In the first step, 60 mg of commercial activated carbon (Vulcan XC‐72) was dispersed in 20 mL of an aqueous solution containing 72.7 mg of CoCl_2_·6H_2_O. The resulting suspension (denoted as mixture A) was subjected to brief ultrasonication for 10 s to disrupt particle agglomeration. During the sonication process, the suspension was simultaneously stirred vigorously with a glass rod to facilitate homogeneous dispersion of the XC‐72 carbon powder within the aqueous medium. Subsequently, mixture A was magnetically stirred for 2 h at room temperature to ensure sufficient adsorption and interaction between Co^2^^+^ species and the carbon support. In the second step, 5.0 g of reducing agent solution (containing 46.2 mg of NaBH_4_, 99%, Sigma–Aldrich Co.) was rapidly dropped in 2 s followed by the injection of 5g Pd precursor in 1 s in the mixture (A). The Pd precursor (100 mM) is the 1.0 M HCl_(aq)_ containing 54.2 mg of PdCl_2_. After that, the mixture was stirred at 400 rpm for 10 min to form solution B. In the third step, 100 µL of water solution containing 0.5 mg of Cu^2+^ was rapidly dropped in the solution B followed by the injection of reduction agent solution containing 100 mg of DI water and 0.5 mg of NaBH_4_. The solution was then stirred for 10 mins to stabilize the samples. For sample decorating with CuO_x_ nanocluster, the concentration of Cu^2+^ precursor and reduction agent is 10‐times to that of CPCu‐1 in the third step. In all synthesis processes, the solution was stirred at 400 rpm for keeping the uniformity of chemical reactions. After reaction, the mixture was centrifuged at 9000 rpm for 15 min and washed by a regular protocol for removing the residual ions (i.e., Na^+^ and Cl^−^). Details for the sample preparation, physical structure inspections and electrochemical characterizations are provided in electronic supplementary information (**ESI**) [34]. Furthermore, to ensure accurate quantification of active elements, the catalyst compositions were determined by ICP‐MS analysis, and the corresponding results are provided in Table S1.

## 3 Results and Discussion

**FIGURE 1:**
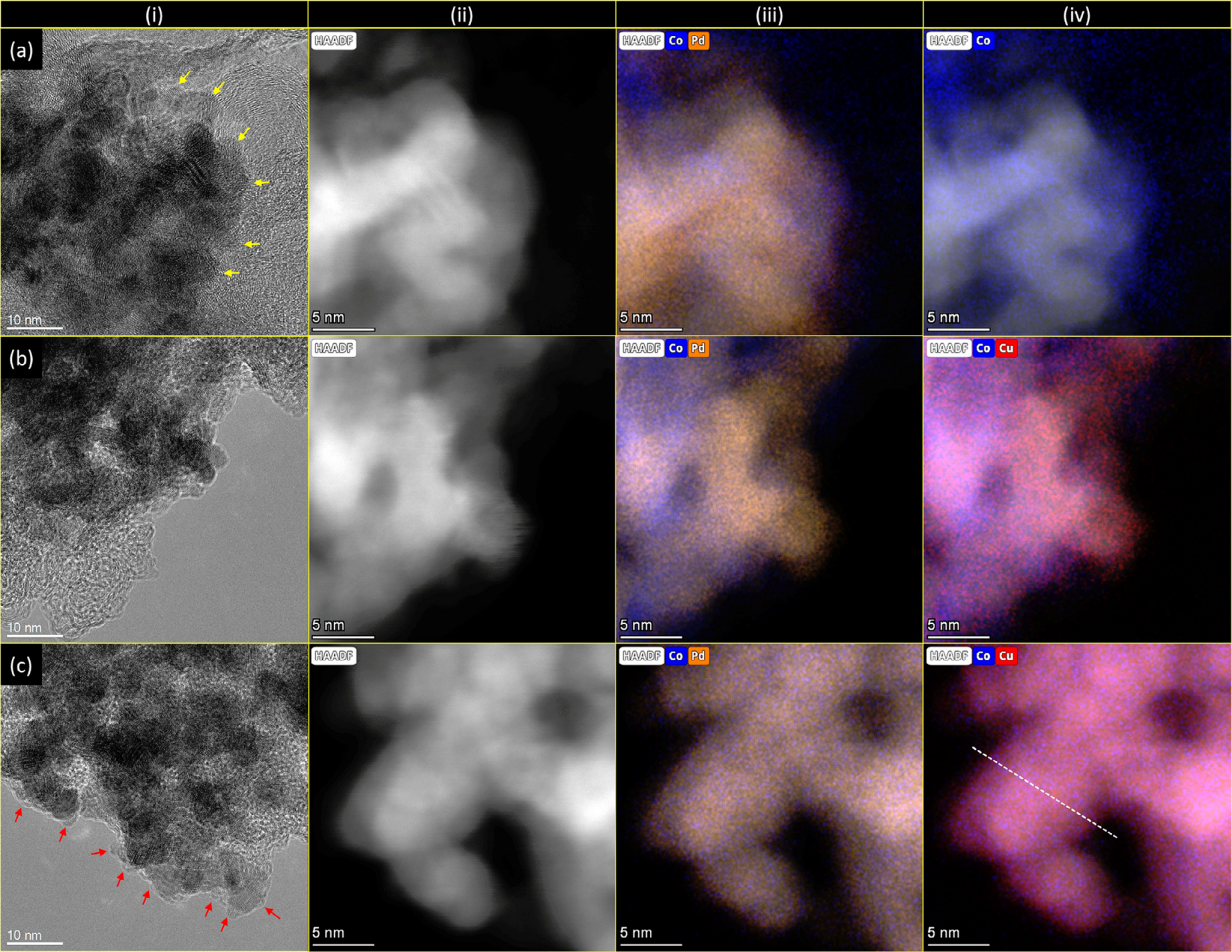
The high‐resolution transmission electromicroscopy (HRTEM) images of (a) CoO@Pd, (b) CPCu‐1 and (c) CPCu‐10 (region (i)). The images of HAADF‐STEM are illustrated in region (ii). The corresponding elementary EDS maps for Co/Pd and Co/Cu are represented in regions (iii) and (iv).

Figure 1 summarizes the electron microscopy analyses of the CoO@Pd, CPCu‐1, and CPCu‐10 catalysts. Panels (i) and (ii) present the corresponding HRTEM and HAADF‐STEM images, respectively, while panels (iii) and (iv) show the elemental EDS maps. From the HRTEM results (Figure 1a‐i), the average particle size of CoO@Pd is determined to be 7–8 nm, and the particles display blurred boundaries and nearly spherical morphology (yellow arrows). Such morphology originates from local coalescence events, where Pd atoms diffusing through the aqueous phase during growth deposit onto adjacent Pd nuclei and induce partial interparticle necking. Upon introducing 1 wt.% Cu decoration (CPCu‐1), the average particle size decreases to 5–7 nm, and the previously observed interparticle necking is largely eliminated. At this stage, Cu atoms effectively suppress uncontrolled Pd‐to‐Pd atomic diffusion, consistent with the appearance of atomic‐scale surface defects and subtle protrusions on individual NPs (Figure 1b‐i). When the Cu loading is further increased to 10 wt.% (CPCu‐10), the particle size again increases to 8–9 nm, and the morphology reverts to a more spherical profile (Figure 1c‐i). Distinct 1–2 nm droplet‐like crystalline domains (red arrows) emerge on the Pd particle surface. This phenomenon aligns well with classical heterogeneous nucleation and crystallization theory. At low coverage, Cu atoms preferentially nucleate on high‐energy Pd surface defects (terraces, point defects) and inter‐faceted regions (edges, corners). Due to the large lattice mismatch between Cu and Pd, Cu nuclei remain in a quasi‐liquid or droplet‐like configuration as long as their size is below the critical crystallization threshold. Once most defect sites are saturated, subsequently deposited Cu atoms grow epitaxially on these nuclei, giving rise to the observed droplet‐like CuO_x_ domains on Pd. These results confirm that Cu atoms are uniformly and atomically dispersed on Pd surfaces at low loading but transition into discrete CuOx nanodomains at higher loading. HAADF‐STEM imaging (Figure 1a‐ii) further reveals that CoO@Pd NPs possess ill‐defined crystal facets and irregular shapes, consistent with the HRTEM observations. After Cu modification at 1 wt.%, CPCu‐1 exhibits truncated surface facets and more distinct interparticle boundaries, indicative of Cu‐induced suppression of Pd aggregation. As Cu loading increases to 10 wt.%, the particles again display a smoother, more rounded morphology, consistent with the HRTEM features. The EDS elemental maps provide complementary structural insights. In CoO@Pd (Figure 1a‐iv), Co exists as sheet‐like structures, while Pd forms aggregated NPs deposited on the surface (Figure 1a‐iii). Relative to CoO@Pd, the smaller particle size in CPCu‐1 confirms that Cu decorating species restrict Pd surface diffusion during nucleation and growth. In contrast, CPCu‐10 exhibits larger Pd NPs and intense Cu signals at the particle perimeter, indicating extensive Cu surface deposition. Line‐scan EDS profiles further clarify the spatial distribution of Pd and Cu. In CPCu‐1 (Figure S1a), Pd exhibits a well‐defined core peak between 3–10 nm, while Cu shows only weak signals overlapping partially with the Pd‐rich region—consistent with light Cu decoration on Pd surfaces. Conversely, CPCu‐10 (Figure S1b) displays strong Cu signals primarily at the outer shell, forming a characteristic Pd@Cu heterostructure. This progressive structural evolution highlights the importance of interfacial electronic coupling and bimetallic synergism. The Pd core provides a conductive metallic scaffold with extended d‐orbitals, while the Cu species—either atomically dispersed (CPCu‐1) or forming surface CuO_x_ clusters (CPCu‐10)—modulate the d‐band center of Pd via electronic transfer and modified coordination environments. Such electronic redistribution optimizes the adsorption energies of oxygenated intermediates, stabilizes key reaction species, and decreases activation barriers, ultimately enhancing catalytic selectivity and activity. A combined analysis of the XRD diffraction peak intensities (indicative of crystallinity) and the full width at half maximum (FWHM, reflecting coherent domain size) for different crystallographic planes (Figure S2) provides clear insight into the structural evolution of Cu species as a function of composition. The results indicate that, in the CPCu‐1 sample, Cu species are predominantly atomically dispersed and uniformly distributed across both the CoOx support and the Pd nanoparticle surfaces, without forming distinct crystalline phases. As the Cu content increases to CPCu‐2, Cu species begin to undergo initial aggregation, giving rise to sub‐nanometric oxide clusters that remain homogeneously distributed on the catalyst surface. At higher loading (CPCu‐10), Cu species further evolve into more stabilized sub‐nanostructures, distributed across the CoOx support and the Pd nanoparticle surfaces and interfaces. This compositional evolution from atomic dispersion to sub‐nanometric clustering is expected to play a decisive role in modulating interfacial active sites and catalytic kinetics, which is further corroborated by the subsequent electrochemical performance analysis.

**FIGURE 2:**
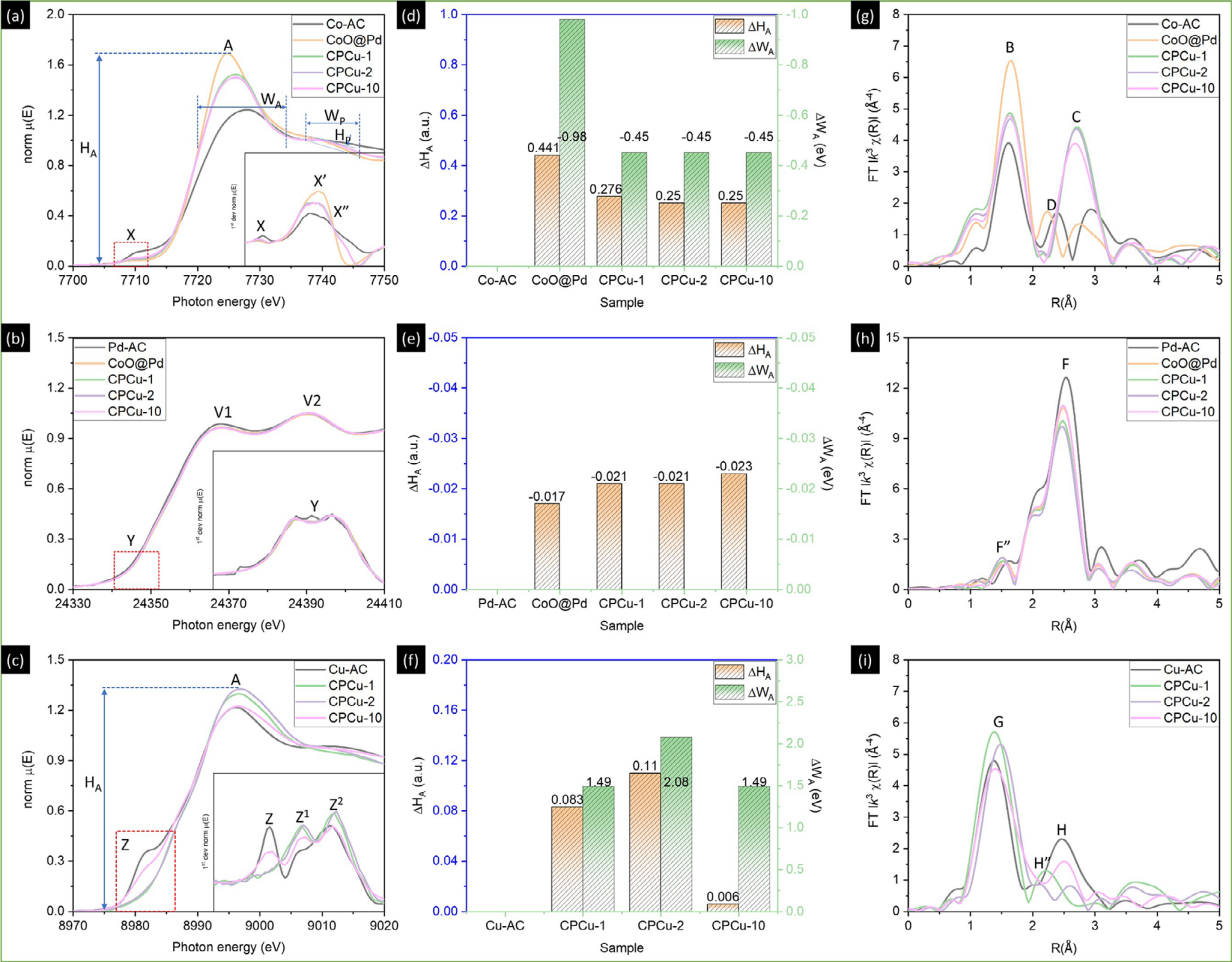
Element‐specific XANES and FT‐EXAFS analysis; (a–c) Normalized XANES spectra at the (a) Co K‐edge, (b) Pd K‐edge, and (c) Cu K‐edge for the reference samples and CPCu series. Insets highlight the pre‐edge and near‐edge features (X, Y, Z regions); (d–f) Quantitative comparison of the variations in absorption height (ΔH_A_) and white‐line width (ΔW_A_) extracted from the respective K‐edge spectra of Co, Pd, and Cu. (g–i) Corresponding Fourier‐transformed EXAFS spectra (|FT χ(k)| vs R) at the (g) Co, (h) Pd, and (i) Cu K‐edges.

Figure 2a shows the Co K‐edge XANES spectra of the experimental catalysts, together with the control and reference samples, while the corresponding first‐derivative curves are displayed in the inset. For a typical 3d transition‐metal K‐edge XANES spectrum, the intensity of peak A scales with the number of empty 4s/4p hybridized states, the full width at half maximum of this peak (**W_A_**) is proportional to the band width, and the ratio of the post‐edge hump height to its width (**H_P_/W_P_**) is positively correlated with the degree of local structural order around the absorber [35]. The pre‐edge feature labeled X corresponds to the quadrupole‐allowed 1s → 3d transition. This feature reflects the centroid position of unoccupied Co 3d states and is therefore highly sensitive to local crystal‐field splitting and 3d hole density. Variations in its intensity and position provide insight into changes in electronic configuration and ligand‐field symmetry. The X′ feature observed in the first‐derivative spectrum represents the maximum slope of the absorption edge and corresponds to the onset of the dipole‐allowed 1s → 4p transition. This feature serves as an effective indicator of oxidation state evolution. The higher‐energy resonance labeled X″ originates from multiple‐scattering contributions and encodes local geometric information, including coordination symmetry and bond‐length distribution. In oxide systems, the intensity of such multiple‐scattering features is often positively correlated with oxygen‐vacancy density due to the symmetry distortion introduced by lattice defects. To facilitate quantitative comparison, the H_A_ and W_A_ values for all samples are summarized in Figure 2d, while the enlarged post‐edge region and H_P_/W_P_ ratios are compiled in Figure S3a. Relative to the Co‐AC reference, CoO@Pd exhibits an increase in H_A_ of 0.441, indicating a substantial enhancement in 4s/4p vacancies at Co sites. This can be ascribed to interfacial electron localization driven by the electronegativity difference and heterogeneous junction between Co and Pd. Upon Cu decoration, ΔH_A_ decreases to 0.276 at 1.0 wt.% Cu and further to 0.25 when the Cu loading exceeds 2.0 wt.%, demonstrating that the CuO_x_ overlayer exerts a steric protection effect, reducing the probability of Co being directly exposed at the surface and thereby partially alleviating its oxidation. The evolution of the 4s/4p band width with Cu content follows the same trend. Compared with Co‐AC, CoO@Pd shows a narrowed W_A_ (ΔW_A_ = –0.98 eV), indicating a pronounced contraction of the Co 4s/4p band in the Co_3_O_4_‐supported Pd NPs system [30, 32]. This band narrowing arises from interfacial electronic restructuring and bonding effects: because Pd is more electronegative than Co, net electron transfer from Co to Pd lowers the outer‐shell electron density at Co sites and enhances the localization of its 4s/4p orbitals. Simultaneously, the formation of Pd‐O‐Co and/or Pd‐Co bonds promotes significant sp–d hybridization, weakening the overlap between Co 4s/4p and Co 3d–O 2p bands and further reducing the band width. In addition, the reducing character of Pd can locally convert Co_3_O_4_ to oxygen‐deficient CoO_x_, generating O_V_s and local lattice strain that distort Co─O─Co bond angles and diminish band delocalization [27]. Altogether, the narrowed Co 4s/4p band reflects interface‐induced electron localization and band de‐overlap, which in turn tailors the Co─O─Pd electronic coupling and underpins the superior interfacial catalytic activity of this system in O_2_ reduction. As the CuO_x_ overlayer thickness increases from 1.0 to 10.0 wt.%, the Pd/CoO_x_ heterointerface becomes progressively encapsulated by a sterically hindering CuO_x_ shell, which suppresses direct Pd‐Co electronic coupling and interfacial charge extraction [30]. This steric protection effect effectively attenuates the electron localization driven by the Pd‐Co electronegativity contrast, relaxes local lattice strain, and improves Co─O─Co orbital overlap, thereby partially restoring the delocalized character of the Co 4s/4p band and slightly increasing its width. This thickness‐dependent band re‐broadening highlights the role of the CuO_x_ layer as an electronic buffer and decoupling layer at the Pd‐CoO_x_ interface, underscoring its potential for interfacial band engineering and catalytic activity modulation. These conclusions are further supported by the first‐derivative spectra (Figure 2a inset), where the decrease in Co near‐edge energy (feature X) and the changes in peak smearing within the 4s/4p hybrid region corroborate the evolution of interfacial band coupling and steric protection effects.

Figure 2b shows the normalized Pd K‐edge XANES spectra of all samples, and the extracted ΔH_A_ and ΔW_A_ parameters are summarized in Figure 2e. At the Pd K‐edge (Figure 2c), the XANES spectra provide additional insight into the electronic structure of Pd nanoclusters following Cu decoration. In the near‐edge region, the weak pre‐edge shoulder labeled Y corresponds to the quadrupole‐allowed 1s → 4d transition, probing unoccupied Pd 4d states. This feature is particularly sensitive to local symmetry distortion and d‐hole density. The nearly identical intensity and energy position of Y across all samples indicate that Pd 4d occupancy remains essentially unchanged upon Cu incorporation, confirming that the Pd metallic core does not undergo significant charge depletion or oxidation. The main absorption maximum V1 arises from the dipole‐allowed 1s → 5p transition and reflects the density of unoccupied Pd 5p states. Because the white‐line intensity scales with the 5p density of states (DOS), the minimal variation in V1 among CoO@Pd and CPCu‐x samples suggests that Pd maintains a predominantly metallic electronic population. This observation effectively excludes substantial Pd → Cu charge transfer or Pd oxidation induced by Cu decoration. The higher‐energy feature V2 is attributed to multiple‐scattering resonances and therefore contains information regarding local geometric structure, particularly Pd─Pd and Pd─O coordination. The nearly invariant line shape and intensity of V2 across the catalyst series indicate preservation of first‐shell Pd coordination. This finding implies that Pd─Cu alloy formation or significant substitution of Pd atoms within the metallic nanoclusters does not occur to an appreciable extent. Instead, Cu species are more plausibly located at the oxide–metal interface rather than incorporated into the Pd lattice. As shown in Figure S4, the near‐edge profiles of all catalysts closely resemble that of metallic Pd foil, unambiguously confirming that Pd species in these materials retain a metallic electronic configuration. As seen in Figure 2e, the slight decrease in H_A_ for CoO@Pd relative to Pd‐AC indicates that Pd in CoO@Pd is even more metallic in nature. This observation is noteworthy because XRD analysis (Figure S5) reveals that CoO@Pd possesses a smaller Pd coherent length and thus a larger specific surface area, which would typically favor increased oxygen adsorption and a higher Pd oxidation state. However, the experimentally observed lower oxidation tendency therefore provides additional evidence for the interfacial charge localization from Co oxides to Pd NPs inferred from the Co K‐edge XANES analysis [32, 36]. With increasing Cu loading, H_A_ decreases progressively, indicating that the growing Cu‐containing overlayer indeed exerts a protective effect on interfacial Pd sites, suppressing electron depletion and stabilizing a metallic Pd state at the Pd‐Co‐Cu oxide interface.

Figure 2c presents the normalized Cu K‐edge XANES spectra of all catalysts, including the Cu‐AC reference, and the extracted ΔH_A_ and ΔW_A_ parameters are summarized in Figure 2f. To further determine the oxidation states of Cu in the experimental samples, the XANES and EXAFS spectra of Cu_2_O, CuO, and Cu‐AC are provided in Figure S6. Comparison with these standards reveals that Cu species in CPCu‐1 and CPCu‐2 exhibit spectral features close to Cu^+^ (≈1+), whereas those in CPCu‐10 gradually shift toward Cu^2+^, consistent with the formation of nanometer‐scale CuO_x_ crystallites at high loading. This indicates that at low concentration, Cu atoms preferentially exist as atomically dispersed clusters embedded on Pd NPs surfaces, while increasing Cu loading to 10 wt.% promotes the development of CuO_x_ domains with mixed valence between +1 and +2 [37]. Changes in the absorption peak intensity further reflect the hybridization behavior between Cu and surrounding atoms. Specifically, relative to Cu‐AC, ΔH_A_ increases from 0.083 to 0.11 as the Cu loading reaches 2 wt.%, indicating a maximum in 4s/4p orbital vacancies at low Cu amounts. At 10 wt.% Cu, ΔH_A_ decreases sharply to 0.006, reflecting attenuation of interfacial hybridization as CuO_x_ grows into independently coordinated oxide nanostructures. Because the pre‐edge and peak shapes remain nearly identical (Figure 2c inset), the Cu valence does not change significantly in this regime; thus, the increased ΔH_A_ mainly arises from strong Cu─O─Pd electronic coupling, which induces interfacial electron localization around Pd. These results reinforce the conclusions drawn from the Pd K‐edge analysis. Within this low‐concentration range, Cu atoms exhibit two distinct sub‐level features (**Z1 and Z2**) in the first‐derivative curves (Figure 2c inset), indicating splitting of hybridized 4s/4p states. Meanwhile, the absence of a pronounced pre‐edge feature confirms the lack of local geometric symmetry, demonstrating that Cu species do not reside within a periodic oxide lattice but instead exist as atomically dispersed Cu─O─M (M = Pd or Co) moieties distributed across Pd NPs and cobalt oxide surfaces. The lower H_A_ value of CPCu‐1 relative to CPCu‐2 further suggests that CPCu‐1 contains more Cu atoms directly embedded into Pd NPs, forming atomic‐scale Cu‐Pd alloy‐like configurations that suppress oxidation upon air exposure. This interpretation is consistent with EXAFS evidence showing the presence of the **H″ peak**, corresponding to Cu─Pd coordination. At 10 wt.% Cu, Cu atoms aggregate to form nanometer‐sized oxide domains, and the sub‐level electronic structure shifts toward that of CuO/Cu_2_O NPs (as shown in the inset), confirming the transition from atomically mixed Cu‐Pd environments to bulk‐like CuO_x_ characteristics [38].

Figure 2g shows the Co K‐edge EXAFS spectra, in which **peaks B** and **C** correspond to Co─O and mixed Co─O/Co─Co scattering in the first and second coordination shells, respectively, while **peak D** arises from metallic Co─Co bonding. Quantitative structural parameters obtained through model fitting are summarized in Table S2. For the Co‐AC reference, the Co─O coordination number (CN = 1.69) and the presence of a weak Co─Co contribution (CN < 0.5) indicate a locally disordered Co oxide matrix containing small metallic Co clusters. These features are consistent with its XANES signature—namely, the suppressed post‐edge hump, enhanced pre‐edge peak, and distinctive H_A_ values (Figure 2a, Figure S3a,c). Relative to Co‐AC, CoO@Pd shows suppression of **peak D** and a pronounced increase in **peak B**, indicating oxidation of metallic Co clusters. This behavior likely results from Pd NPs incorporation into the CoO_x_ matrix, which exposes previously embedded metallic Co to the surface, rendering it susceptible to oxidation. This is supported by the increased Co─O coordination number (CN = 3.54). In Cu‐modified samples, the Co oxidation state does not significantly differ from that in CoO@Pd (as verified by peak X in Figure 2a inset), but the structural ordering improves (Figure S3c). The simultaneous suppression of **peak B** and enhancement of **peak C** reflect a reduction in Co─O first‐shell coordination (CN = 2.7) accompanied by emergence of a second‐shell Co─O contribution (CN = 1.57). These features suggest that Cu decoration transforms the Co environment into a structure resembling Co(OH)_2_‐like coordination, as supported by comparison with the reference spectrum (Figure S7). However, the substantially weaker intensities of peaks B and C (relative to Co(OH)_2_) indicate that all Cu‐containing samples possess a high density of O_V_s around Co sites (O_V_(Co)). For CPCu‐10, peak C is slightly weakened and shifted to lower R, consistent with steric protection introduced by thick CuO_x_ layers, which create Co deficiency at the Cu─Co interfacial region. EPR analysis (Figure S8) confirms that CPCu‐1 exhibits the highest oxygen‐vacancy concentration among all samples. This behavior is consistent with the presence of a high proportion of Cu in Co─O─Cu linkages, where charge compensation drives the formation of Co‐site defects. Together, the Co K‐edge EXAFS data and EPR results demonstrate a strong interplay between Cu incorporation, O_V_ formation, and Co coordination restructuring.

Figure 2h presents the Pd K‐edge EXAFS spectra, where radial peaks F and F″ correspond to metallic Pd‐M (M = Co or Cu) scattering and Pd─O scattering originating from adsorbed oxygen, respectively. Based on peak assignment, the Pd‐AC reference consists of metallic Pd NPs with a Pd─Pd coordination number of 8.02, together with a measurable degree of surface oxygen adsorption (Pd─O CN = 0.86). Compared with Pd‐AC, the CoO@Pd sample exhibits both suppression and a leftward shift of peak F, reflecting a decrease in Pd─Pd coordination number to 6.92 and a contraction of the Pd─Pd bond distance to 2.739 Å. The reduced CN indicates a shorter coherent length, while the shorter bond distance is indicative of atomic‐scale defects within the Pd NPs. These defects emerge because Pd nucleates and grows rapidly on the Co oxide support—which has a relatively high lattice mismatch—before sufficient surface relaxation can occur, resulting in strained and defect‐rich Pd domains. When Cu is introduced, the intensity of peak F decreases further, reaching its minimum at CPCu‐2, corresponding to a Pd‐Pd CN of 6.61, which confirms that low Cu loadings (≤2 wt.%) embed directly into the Pd surface and generate additional structural defects. In contrast, increasing the Cu loading to 10 wt.% results in a pronounced increase in peak F intensity, with Pd‐Pd CN recovering to 7.38. This reversal indicates that once the CuO_x_ overlayer becomes sufficiently thick, a steric protection effect emerges, reducing direct oxidation or disordering of the Pd surface and thereby increasing atomic ordering around Pd atoms. The presence of Pd‐Cu bonding at low Cu concentrations is further supported by the detection of a small but non‐negligible Pd‐Cu coordination number (CN = 0.45), demonstrating Cu incorporation at the Pd interface. Meanwhile, the protective role of CuO_x_ is validated by the reduction of the Pd‐O coordination number at higher Cu loadings, confirming decreased adsorption of oxygen on the Pd surface.

Figure 2i shows the Cu K‐edge EXAFS spectra, which include radial peaks G, H, and H″ arising from Cu─O, Cu─O (higher‐shell), and metallic Cu─Cu scattering, respectively. According to peak assignment and structural parameters (Table S2), the Cu‐AC reference contains defective CuO_x_ NPs, as evidenced by first‐ and second‐shell Cu─O coordination numbers of 2.56 and 2.58 at R = 1.878 and 2.895 Å, respectively. The second‐shell Cu─Cu CN of 0.63, far below that of crystalline Cu_2_O (Table S2), confirms the presence of substantial structural disorder around Cu sites. Compared with Cu‐AC, peak G intensifies significantly in CPCu‐1, indicating an increase in first‐shell Cu─O coordination to CN = 3.54 at R = 1.884 Å. This strong enhancement aligns with the ΔH_A_ trend (Figure 2f) and reflects pronounced heteroatomic mixing between Cu, Pd, and Co. However, clear deviation of the first‐derivative XANES peak shape from that of Cu_2_O (Figure 2c inset) confirms that Cu does not reside in a periodic oxide lattice. Model fitting reveals that peak H″ originates from Cu‐Pd (CN = 1.07 at 2.576 Å) and Cu─Cu (CN = 0.88 at 2.948 Å) interactions, demonstrating that a significant fraction of Cu atoms embeds directly into Pd surfaces to form mixed‐metal Cu_2_Pd clusters, characterized by coexisting metallic and oxidized Cu environments. When the Cu loading increases to 2.0 wt.% (CPCu‐2), peak G decreases in intensity and shifts to higher R, corresponding to a reduction in Cu─O CN to 3.09 and an increase in bond length to 1.95 Å. The second coordination shell nearly disappears, and the pre‐edge amplitude increases slightly (Figure S3d), indicating that Cu atoms increasingly aggregate into small CuO_x_ clusters on Pd surfaces, thereby diminishing metallic Cu‐Pd interactions. At 10 wt.% Cu (CPCu‐10), peak G continues to weaken while peak H becomes more pronounced. The first‐shell Cu─O CN decreases to 2.69, with the bond distance reverting to 1.908 Å, close to Cu_2_O, indicating partial crystallization of CuO_x_. Meanwhile, the second‐shell Cu─O CN increases to 1.51 at R = 3.276 Å, confirming the formation of nascent CuO_x_ nanocrystals. The hump between peaks H and H″ corresponds to Cu─O─Pd interfacial coordination (CN = 0.51 at 2.848 Å), demonstrating the coexistence of oxide and metal bonding at the Cu─Pd interface. Altogether, the EXAFS results indicate that CuO_x_ grows into 1–2 nm nanocrystals at high Cu loading, with Cu oxidation states transitioning between Cu^+^ and Cu^2^^+^—consistent with XANES observations (Figure S6a inset) [39]. Finally, the electronic interactions and structural transformations among Co, Pd, and Cu atoms inferred from EXAFS are further validated by the corresponding WT‐EXAFS contour maps (Figures S9–S11) and PFY‐XANES spectra (Figure S12), which reveal consistent trends in scattering peak evolution, atomic coordination restructuring, and heterointerface formation.

**FIGURE 3:**
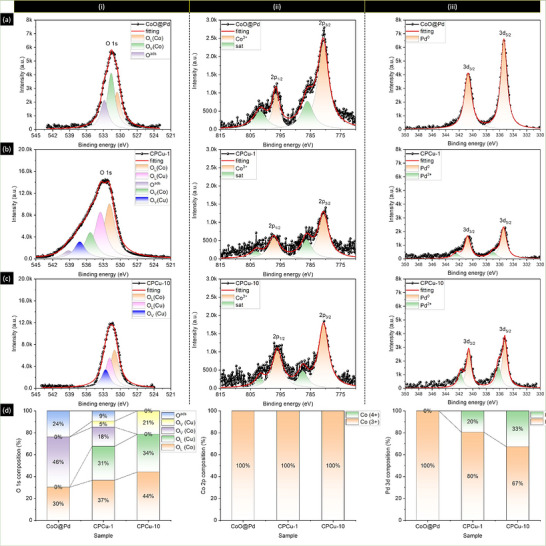
XPS spectra and fitting curves of (a) CoO@Pd, (b) CPCu‐1, and (c) CPCu‐10. The spectra at O1s, Co 2p, and Pd 3d orbitals are shown in columns (i), (ii), and (iii), respectively. Corresponding fitting results are shown in 4(d)‐i to 4d‐iii.

Figure 3a‐i presents the O 1s XPS spectrum of CoO_x_‐supported Pd NPs (CoO@Pd). The spectrum can be deconvoluted into three oxygen species assigned to lattice oxygen in cobalt oxide (O_L_(Co)), O_V_(Co), and surface‐adsorbed oxygen species (*O^ads^). Quantitative fitting yields relative contributions of 30% O_L_(Co), 46% O_V_(Co), and 24% *O^ads^, indicating that the CoO_x_ framework is highly oxygen‐deficient. This observation is consistent with the Co K‐edge EXAFS results, which reveal substantial O_V_ formation and a disordered Co─O coordination environment. Figure 3b‐i shows the O 1s spectrum of the CPCu‐1 catalyst. In contrast to CoO@Pd, five distinct oxygen species are required to adequately fit the spectrum: O_L_(Co), O_L_(Cu), O_V_(Co), O_V_(Cu), and *O^ads^; where O_L_(Cu) and O_V_(Cu) respectively denote the lattice oxygen and O_V_ in the CuO_x_. Their relative proportions, summarized in Figure 3d‐i, are: O^ads^: 9%; O_V_(Cu): 5%; O_V_(Co): 18%; O_L_(Cu): 31%; O_L_(Co): 37%. Although Cu accounts for only ∼1 wt.% of the total mass, the unusually high fractions of O_L_(Cu) and O_V_(Cu) indicate strong surface enrichment of Cu species near the outer surface of Pd NPs. This surface‐localized Cu gives rise to abundant Cu‐O lattice oxygen and Cu‐centered vacancy sites, both of which act as electronically flexible centers capable of regulating interfacial charge redistribution [37]. Figure 3c‐i displays the O 1s spectrum of CPCu‐10. Owing to the high Cu loading (10 wt.%), the surface oxygen chemistry simplifies to three dominant components: O_L_(Co), O_L_(Cu), and O_V_(Cu). Quantitative analysis reveals relative contributions of: O_L_(Co): 44%; O_L_(Cu): 34%; O_V_(Cu): 21%. At this loading, the CuO_x_ overlayer on CoO@Pd becomes thicker than 1–2 nm, comparable to or exceeding the effective XPS probing depth. As a result, Co‐related O_V_(Co) signals and *O^ads^ contributions are strongly attenuated or fully obscured. The complete CuO_x_ overlayer also exerts a pronounced steric protection effect, inhibiting O_V_ formation in the underlying CoO_x_ and suppressing exposure of reactive Co surface sites. Although O_V_(Cu) remains detectable and represents an active center capable of initiating the oxygen reduction reaction (ORR), the encapsulating CuO_x_ layer restricts interfacial oxygen mobility and electron–mass transport coupling. Thus, despite the higher concentration of Cu, the CPCu‐10 catalyst does not show further improvement in ORR activity (Figure 6). Instead, the excessively thick CuO_x_ layer becomes transport‐limiting, diminishing the accessibility of active sites and restricting the beneficial interfacial Pd─Cu─Co electronic interactions observed at lower Cu contents.

Figure 3a‐ii,b‐ii,c‐ii presents the XPS Co 2p spectra of CoO@Pd, CPCu‐1, and CPCu‐10, respectively, with the corresponding quantitative fitting results summarized in Figure 3d‐ii. In all cases, cobalt species are found exclusively in the **Co^3^^+^ oxidation state (100%)**, with no detectable Co^2^^+^ contribution. The stabilization of surface Co^3^^+^ across all catalysts can be attributed to three strongly coupled mechanisms: interfacial charge transfer, vacancy‐driven reoxidation, and strain‐ and confinement‐induced electronic restructuring [27, 30, 36]. At the Pd/CoO_x_ or CuO_x_/CoO_x_ interfaces, a substantial degree of electronic polarization is generated due to the Fermi‐level mismatch between the oxide and the adjacent metallic layers. Electrons are transferred from Co 3d orbitals into Pd or Cu sites with higher work functions, depleting electron density within the CoO_x_ lattice. This electron withdrawal enhances the ionic character of Co─O bonds and stabilizes surface Co^3^^+^‐O^2^^−^ dipoles, effectively shifting Co toward a higher oxidation state. Additionally, the abundant O_V_s identified through EXAFS and O 1s XPS analyses enable a defect‐mediated reoxidation pathway, in which mobile oxygen species—either diffusing through the CoO_x_ lattice or supplied by CuO_x_‐derived lattice oxygen—repopulate vacancy sites. This process locally oxidizes Co^2^^+^ → Co^3^^+^ and generates a dynamic mixed‐valence environment. The vacancy–oxygen exchange mechanism is further strengthened by the electronic activity of Cu‐modified surfaces, which promote oxygen migration and charge redistribution. A third contributing factor is lattice mismatch–induced strain between the CoO layer and the underlying Pd or Cu‐containing phases. Compressive strain increases Co 3d–O 2p orbital overlap and shifts electron density toward oxygen, driving the Co cations into an effectively Co^3^^+^‐like electronic configuration. When the CoO layer becomes ultrathin (approaching the sub‐nanometer regime), quantum confinement effects further narrow the Co 3d bandwidth, lower the Co 3d energy level relative to the Fermi level, and thermodynamically favor the stabilization of high‐valence Co species. Through these synergistic mechanisms (interfacial electron transfer, vacancy‐mediated redox cycling, and strain/confinement‐induced electronic restructuring) the surface Co^3^^+^ species become thermodynamically stabilized, and such high‐valent cobalt centers are well known to enhance redox‐driven catalytic pathways central to oxygen‐involved electrochemical reactions [40]. In addition to Co valence‐state evolution, the Co 2p XPS spectra show a systematic decrease in overall photoemission intensity from CoO@Pd to CPCu‐1 and CPCu‐10. This attenuation arises primarily from photoelectron shielding by the CuO_x_ overlayer, confirming that Cu species preferentially accumulate near the catalyst surface and partially encapsulate the underlying CoO_x_‐Pd composite. At 10 wt.% Cu, the Co 2p signal partially recovers and becomes more intense than that of CPCu‐1, indicating that the CuO_x_ overlayer becomes non‐uniform at higher Cu loadings. This transition reflects the morphological evolution of CuO_x_ from atomically dispersed species at low loading to 1–2 nm island‐type clusters at higher loading—consistent with HRTEM observations in Figure 1c‐i. Together, these results demonstrate that: (1) surface Co^3^^+^ is universally stabilized across all catalysts through interface‐ and defect‐driven electronic mechanisms, and (2) CuO_x_ spatial distribution shifts with loading, transitioning from a conformal, ultrathin modification layer at low Cu content to a discontinuous cluster‐type overlayer at high Cu content.

Figure 3a‐iii,b‐iii,c‐iii shows the Pd 3d XPS spectra of CoO@Pd, CPCu‐1, and CPCu‐10, respectively, with the corresponding quantitative results summarized in Figure 3d‐iii. For the CoO@Pd sample, Pd exists exclusively in the metallic state (Pd^0^, 100%), indicating that the Pd NPs are effectively encapsulated by the surrounding CoO_x_ domains. This configuration suppresses Pd─O bond formation and prevents the adsorption of oxygen species, yielding a purely metallic Pd 3d signature. In contrast, CPCu‐1 exhibits a mixed‐valence Pd population, comprising 80% Pd^0^ and 20% Pd^2^^+^. The emergence of Pd^2^^+^ suggests that Cu incorporation induces partial oxidation of surface Pd atoms through interfacial Pd─O─Cu bonding or the formation of an ultrathin PdO_x_ shell [40]. This behavior is consistent with the strong oxidative character of Cu─O moieties, which can withdraw electron density from neighboring Pd sites, thereby driving the Pd^0^ → Pd^2^^+^ transition. When the Cu loading increases to 10 wt.% (CPCu‐10), the Pd^2^^+^ fraction further rises to 33%, reflecting an enhanced degree of Pd oxidation. At this loading, the CuO_x_ overlayer reaches a thickness approaching several nanometers (<3 nm), creating a more oxidizing surface environment. The higher density of Cu^2^^+^/Cu^+^ species and associated O_V_s promotes oxygen migration and spillover, which intensifies interfacial oxidation at the Pd‐CoO_x_ boundary and increases the population of Pd^2^^+^. A systematic decrease in Pd 3d photoemission intensity is observed from CoO@Pd → CPCu‐1 → CPCu‐10, paralleling the trend revealed in the Co 2p spectra. This attenuation arises from photoelectron shielding by the CuO_x_ overlayer, confirming that Cu species preferentially accumulate at or near the catalyst surface. Interestingly, CPCu‐10 displays a slightly higher Pd 3d signal intensity than CPCu‐1. This suggests that, at elevated Cu loading, the CuO_x_ layer becomes non‐uniform, forming 1–2 nm island‐type clusters rather than a continuous coating. The incomplete surface coverage reduces shielding efficiency and allows a greater fraction of Pd photoelectrons to escape. This morphological evolution (from atomic Cu decoration (CPCu‐1) to nanocluster‐type CuO_x_ domains (CPCu‐10)) is fully consistent with the HRTEM observations in Figure 1c‐i. These results collectively confirm that Cu initially disperses at the atomic scale on Pd NP surfaces but transitions to localized CuO_x_ island growth as the Cu concentration increases [41].

**FIGURE 4:**
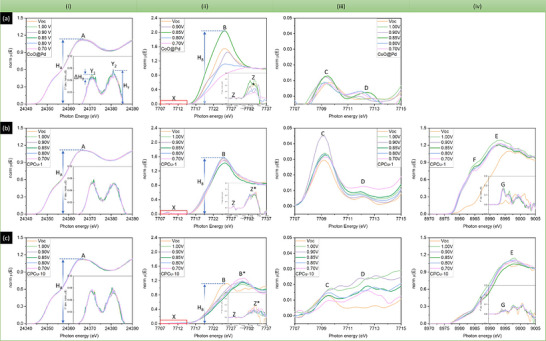
PFY‐XANES spectra of (a) Co@Pd, (b) CPCu‐1, and (c) CPCu‐10 under potential driven conditions in ORR. The spectra at Pd K‐edge, Co K‐edge, and Cu K‐edge are demonstrated in column (i), (ii), and (iv), respectively. The enlarged region X in the column (ii) is shown in region (iii).

Figure 4a–c displays the operando PFY‐XANES spectra of CoO@Pd, CPCu‐1, and CPCu‐10 acquired under ORR‐relevant potentials between V_oc_ and 0.70 V vs. RHE. Columns (i), **(ii)**, and **(iii),** respectively present the spectra collected at the Pd, Co, and Cu K‐edges. Figure 4(a‐i) presents the Pd K‐edge XANES spectra of CoO@Pd acquired under a series of applied potentials. The main absorption peak A corresponds to the 1s → 4p transition of Pd, and its intensity (H_A_) reflects the unoccupied Pd 4d‐4p density of states. As the potential decreases toward 0.70 V, H_A_ exhibits a slight attenuation, indicative of the gradual reduction of surface Pd sites from Pd^2^^+^ to metallic Pd^0^. This reduction is accompanied by interfacial charge relocation toward the CoO_x_ support. The inset shows the first‐derivative spectra, which provide enhanced sensitivity to subtle edge features. The pre‐edge feature Y_1_ (≈24367 eV) originates from the Pd‐O/Co hybridized 1s → 4d transition, and its energy shift (ΔE(Y_1_)) serves as a descriptor of the Pd oxidation state. The Y_2_ feature (≈24378‐24380 eV), assigned to the 1s → 4p dipole transition, reports on changes in local coordination symmetry and slight modulation of Pd─O bond lengths. Upon stepping the potential from 1.00 to 0.70 V, both Y_1_ and Y_2_ remain nearly invariant in energy with only minor reductions (∆H_Y_) in intensity (H_Y_). These trends indicate a mild redistribution of electron density at the Pd/CoO_x_ interface under oxidative ORR conditions, along with a decrease in surface Pd─O species. Such behavior is characteristic of metallic Pd catalysts in which only surface atoms participate in O_2_ dissociation, while the underlying Pd lattice remains structurally intact. The subtle 4p‐state modulation further confirms that Pd atoms in CoO@Pd function primarily as O_2_‐splitting sites. This assignment is independently validated by operando EXAFS analysis (Figure S13), which shows no perturbation of the Pd–Pd coordination shell. In contrast, the Pd K‐edge spectra of CPCu‐1 and CPCu‐10 (Figure 4b‐i,c‐i) remain completely unchanged—both in edge position and spectral line shape—regardless of the applied potential. These static features unambiguously indicate that Pd does not undergo redox transitions in the CuO_x_‐decorated systems and therefore does not directly participate in O_2_ activation in these catalysts.

Figure 4(a‐ii) shows the potential‐dependent Co K‐edge PFY spectra of CoO@Pd. The main‐edge peak B, whose height (H_B_) scales with the number of 4s/4p holes, serves as a sensitive descriptor of electron redistribution at Co sites. Relative to the open‐circuit condition, a slight enhancement of H_B_ at 1.00 V (without any shift in the edge inflection point) suggests that *O^ads^ migrates from adjacent Pd sites toward Co, increasing Co‐centered ligand‐to‐metal charge interactions without inducing a formal valence change. When the potential is lowered to 0.90 V, H_B_ decreases substantially and the absorption envelope narrows. These features indicate the reduction of *O^ads^ by electrode‐supplied electrons, resulting in increased local structural order and partial reduction of the CoO_x_ matrix. The progressive sharpening of the edge profile reflects the tendency of CoO_x_ to evolve toward a more metallic‐like state. At 0.85 V vs RHE, H_B_ reaches its maximum intensity while the bandwidth becomes the broadest among all tested potentials. This scenario corresponds to the highest density of 4s/4p vacancies and the widest energy distribution of the Co conduction band. Importantly, this potential coincides with the maximum kinetic ORR current (*J*_k_), implying that severe adsorption–desorption dynamics and oxygen relocation induce significant long‐range disorder in CoO_x_. Such disorder increases band dispersion and facilitates rapid electron‐oxygen coupling, enabling Co sites to assume a dominant role during ORR turnover. At 0.80 V, the spectrum shifts further toward metallic Co, with absorption peak B nearly disappearing. This suggests that as the reaction approaches the diffusion‐limited regime, vacancies in Co 4s/4p orbitals diminish rapidly, consistent with reduced participation of Co in O─O bond scission and O‐reduction steps at high current densities. Finally, at 0.70 V vs RHE, H_B_ exhibits an even greater decline. Because the Co oxidation state remains unchanged (as evidenced by the constant energy position of peak Z in Figure S13a), the pronounced decrease in H_B_ signifies that electron donation from the electrode becomes strongly localized at Co sites [40]. Under these highly reducing conditions, Co atoms function as the primary active centers for both O_2_ dissociation and subsequent O reduction, overtaking the role of Pd at low operating potentials.

To resolve the evolution of Co 3d electronic states, the pre‐edge region (region X) is magnified in Figure 4(a‐iii). Peaks C and D arise from quadrupole‐allowed multiple‐scattering transitions involving 1s → 4s/4p excitation coupled with interactions between the Co 4s/4p continuum and the 3d‐derived e_g_ and t_2_g sub‐orbitals. The positions and intensities of these features scale with changes in orbital energy splitting and electron density within the respective sub‐levels. Because the Co valence remains constant across all applied potentials, variations in the pre‐edge features originate solely from modifications in Co─O bonding symmetry, which alter both the e_g_/t_2_g electron density distribution and the corresponding sub‐orbital energies. Simultaneously, shifts in the 4s/4p continuum reflect potential‐dependent chemical shifts and hybridization strength between Co and *O^ads^ species, confirming that Co acts as an active center for O_2_ dissociation. At open‐circuit voltage (V_oc_), the e_g_‐related contribution exhibits the broadest energy distribution but the lowest peak intensity, indicating that the electron density is strongly biased toward the e_g_ manifold. Such spectral characteristics correspond to a distorted tetrahedral Co─O coordination environment enriched with atomic‐scale defects. Upon decreasing the potential to 0.90 V, the emergence of clearly separated C and D peaks signifies that the coordination number of oxygen increases to approximately six, forming a distorted octahedral CoO_6_ environment around the Co centers. Further decreasing the potential to 0.80 V yields the narrowest C‐D peak distribution and the highest peak intensity among all conditions. This indicates that Co experiences the greatest degree of O coordination and substantially weakened Co–*O^ads^ bond strength. These spectral behaviors suggest that *O^ads^ becomes increasingly stabilized at Co sites, reducing electron back‐donation into the metal center, increasing the activation barrier of ORR intermediates, and correlating strongly with the experimentally observed maximum *J*_k_. When the potential is decreased to 0.70 V vs RHE, the slight decrease in the D peak intensity indicates strengthened Co─O interactions due to the accumulation of excess *O^ads^. This phenomenon reflects the growing dominance of *O^ads^ binding at reduced potentials. Taken together, these observations show that both Co and Pd atoms in the CoO@Pd catalyst serve as active centers for O_2_ activation (adsorption and splitting), after which the resulting *O^ads^ species migrate to neighboring atomic sites to regenerate active centers for subsequent ORR turnover. The binding strength of *O^ads^ at oxygen‐vacancy‐associated sites ultimately dictates the rate of O─O bond reduction. Below 0.80 V, substantial electron accumulation at Co sites drastically weakens *O^ads^ binding energy, promoting *O^ads^ recombination pathways that suppress ORR kinetics. This mechanistic insight explains the observed decline in reaction rate at low operating potentials despite the availability of abundant *O^ads^ species.

The PFY‐XANES spectra at the Pd K‐edge (Figure 4(b‐i)) remain invariant in both line shape and intensity (∆H_Y_ = 0) across all applied potentials, indicating that Pd in the CPCu‐1 catalyst does not undergo any detectable valence change. This spectral invariance confirms that Pd sites do not participate in the O_2_ activation step in the CuO_x_‐decorated system. Figure 4(b‐ii) displays the Co K‐edge PFY‐XANES spectra of CPCu‐1 from V_oc_ to 0.70 V vs RHE, together with corresponding first‐derivative curves (**inset** and Figure S13b). The inflection point (peak Z) reveals a slight decrease in Co valence at 0.90 V relative to V_oc_ and 1.00 V, which reverts to the original value when the potential is lowered to 0.85 V. Importantly, only subtle changes are observed in region Z*, confirming that—analogous to Pd‐Co does not serve as an active O_2_‐splitting center in CPCu‐1 during ORR. Variations in the Co K‐edge intensity (H_B_) therefore reflect only the modulation of 4s/4p hole density arising from electron donation from the electrode. Compared with V_oc_, the applied potential leads to a systematic decrease in H_B_, indicating a reduction in 4s/4p vacancies due to increased electron density around Co. This trend reveals strong charge localization at Co sites induced by neighboring O_V_s, which possess high electronegativity and act as efficient electron traps. At the potential corresponding to the highest *J*_k_, H_B_ reaches a minimum, demonstrating that ORR initiation indeed occurs at O_V_ sites, where the density of *O^ads^ and the rate of O_2_ dissociation are maximal. Upon lowering the potential to 0.80 V, H_B_ returns to its initial magnitude. This recovery indicates a dynamic balance between (i) O_2_ splitting at O_V_ sites, which consumes electrons, and (ii) rapid transfer of *O^ads^ to adjacent metal atoms, which regenerates vacancies. At 0.80 V, the rates of these two processes are matched closely, preventing excessive localization of electrons at Co sites and keeping the 4s/4p hole density unchanged. At 0.70 V, H_B_ decreases again because the O_2_ dissociation rate surpasses the capacity of neighboring atoms to remove *O^ads^, leading to the accumulation of excessive *O^ads^ species on the surface. Pre‐edge features (Figure 4(b‐iii)) further elucidate the evolution of local Co coordination. The dominant intensity of peak C over peak D at V_oc_ reflects a predominantly four‐coordinate, tetrahedral Co─O environment, consistent with abundant O_V_s; this is corroborated by the strong long‐pair EPR signal (Figure S8). When the applied potential approaches the maximum *J*_k_, both peaks C and D increase in intensity and slightly narrow in distribution, indicating enhanced 3d electron population and the formation of relatively weaker, more covalent Co‐*O^ads^ bonds. As the potential reaches 0.90 V, peak C increases markedly while peak D returns to its original intensity, reflecting a fully tetrahedral Co─O arrangement in which *O^ads^ generated at Co─O_V_ sites is quickly scavenged by neighboring metal atoms. When the potential is further reduced to 0.85 V, the rate of *O^ads^ formation increases, leading to a gradual rise in Co coordination number and a shift of the pre‐edge signature back toward the original mixed‐coordination state—consistent with the concurrent changes observed in the 4s/4p vacancy density. Figure 4(b‐iv) presents the Cu K‐edge PFY‐XANES spectra. At V_oc_, the strong absorption and energy position indicate that Cu exists predominantly in a highly oxidized state with significant O_V_─Cu bonding, leading to reduced structural symmetry and substantial electron localization near O_V_ sites (see also Figure S12(i)). At 1.00 V, the absorption peak intensity increases sharply, demonstrating that Cu forms direct bonds with reaction‐generated O species. This increases 4s/4p hole density and drives hybridization toward a higher Cu─O coordination environment enriched with *O^ads^. The pronounced downward shift of both the pre‐edge and main‐edge energies indicates a reduction in Cu oxidation state due to rapid *O^ads^ formation and strong coupling with ORR intermediates. These energy shifts also reflect substantial modifications to Cu 3d and 4s/4p orbital energies arising from interfacial coupling among O_V_, Co, and Pd sites. Such orbital stabilization promotes steep local electron‐density gradients that accelerate *O^ads^ migration to reactive sites, thereby enhancing ORR reaction rates and overall catalytic activity.

The PFY‐XANES spectra at the Pd K‐edge (Figure 4(c‐i)) remain unchanged across all applied potentials, confirming that the Pd sites in CPCu‐10 do not undergo valence transitions and therefore do not participate in the O_2_ activation step [32]. Figure 4(c‐ii) shows the Co K‐edge PFY‐XANES spectra of CPCu‐10 from V_oc_ to 0.70 V vs RHE. In the corresponding first‐derivative spectra (inset and Figure S13c), the pre‐edge inflection point (Z), associated with the 1s → 3d transition, nearly disappears at all potentials. The loss of the Z feature reflects a substantial suppression of 3d‐4p hybridization and increased electronic localization at Co sites. This trend indicates a shift from a mixed‐valence Co^2^^+^/Co^3^^+^ oxide environment toward a more metallic or reduced Co configuration, accompanied by weakened Co─O covalency, O_V_ formation, and partial detachment of surface *O^ads^ species. Thus, the diminished pre‐edge intensity marks the electronic activation of Co, where localized 3d states evolve into more delocalized bands capable of injecting electrons into adsorbed intermediates. Similar to region Z, the attenuation and broadening of the Z* region further support the emergence of heterogeneous Co─O─Cu bonding motifs and multiphase structural domains. These features originate from extensive CuO_x_ modification on CoO@Pd, which introduces (i) complex Co─O─Cu electronic hybridization that broadens the Co orbital energy distribution and (ii) steric protection that reduces the oxidation degree of Co, burying a fraction of metallic Co at interfacial regions. As the applied potential enters the ORR‐active regime (1.00 → 0.70 V vs RHE), peak B shifts to higher energy (B*) and broadens, indicating an upward chemical shift of the Co 4s/4p orbitals. This behavior demonstrates that Co participates in O_2_ dissociation, forming Co─O bonds under reaction conditions. When the potential approaches the diffusion‐limited region (0.70 V vs RHE), the peak B* intensity reaches its maximum, implying that accumulated *O^ads^ eventually forms strong, saturating Co‐*O^ads^ interactions that block active sites. This observation aligns well with the intrinsically lower ORR activity of CPCu‐10. The pre‐edge region (Figure 4(c‐iii)) contains only a single peak (C) at V_oc_, consistent with a tetrahedral CoO_4_ environment. Under ORR potentials, peak D progressively emerges and broadens the pre‐edge envelope, indicating increasing Co─O bond strength. Below 0.85 V, peak D becomes more clearly resolved, reflecting the formation of a more ordered local Co─O coordination environment—contrasting sharply with CPCu‐1, whose spectra show distinct tetrahedral–octahedral evolution. The more diffuse spectral features in CPCu‐10 reveal that Co resides within a highly heterogeneous Cu─O─Co hybridized network rather than a well‐defined oxide framework. Figure 4(c‐iv) presents the Cu K‐edge PFY‐XANES spectra. First‐derivative analysis (**inset** and Figure S13c) confirms that the Cu valence remains unchanged at all potentials. At 1.00 V, the pre‐edge feature G reaches its minimum intensity while the main absorption peak E reaches its maximum. This behavior indicates strong Cu─O^ads^ interactions that localize electrons near *O^ads^, increase 4s/4p hole density, and broaden the Cu 3d band—producing the lowest multiple‐scattering intensity among all conditions. As the potential is lowered to 0.85 V, peak E reaches a minimum, illustrating that at the highest reaction current, *O^ads^ is rapidly transferred to neighboring O_V_ and Pd sites, decreasing 4s/4p vacancy density at Cu. In the diffusion‐limited regime (0.80‐0.70 V), peak E returns to the V_oc_ value because *O^ads^ is generated faster than it can be removed, temporarily confining electron density around Cu and regenerating the 4s/4p vacancies. Taken together, the near‐edge evolution at all three elemental edges shows that, in CPCu‐10, Co serves as the primary O_2_ activation site, while adjacent O_V_ and Cu centers capture *O^ads^ to regenerate Co active sites. However, the relatively slow *O^ads^ transfer and reduction kinetics cause *O^ads^ accumulation around Co and Cu, which limits active‐site turnover and explains the lower ORR activity of CPCu‐10 relative to CPCu‐1.

**FIGURE 5:**
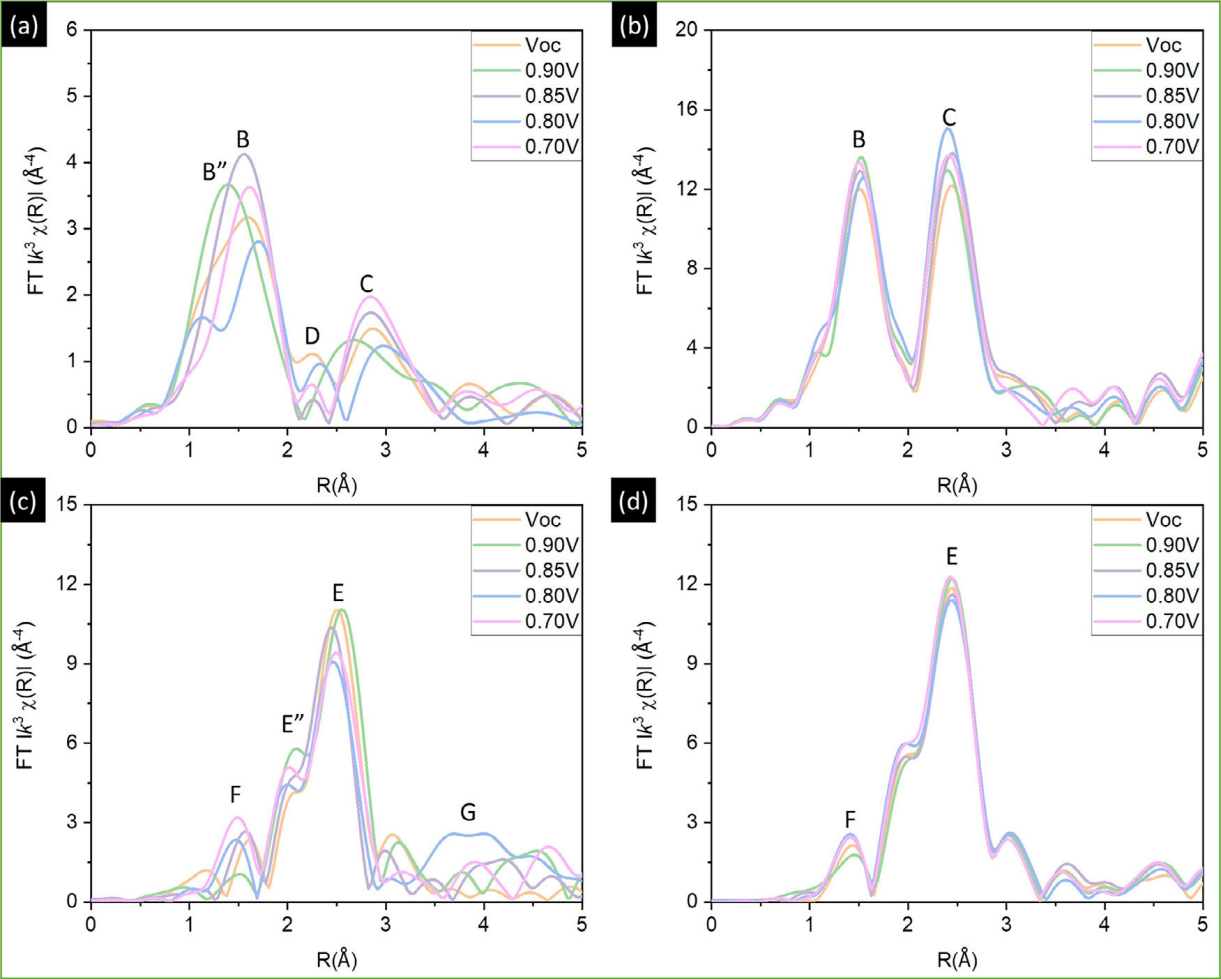
FT‐EXAFS spectra of (a) CoO@Pd and (b) CPCu‐1 at Co K‐edge. The corresponding FT‐EXAFS spectra at Pd K‐edge are demonstrated in (c) and (d). These spectra were collected at the potential driven conditions of ORR.

Figure 5a presents the FT‐EXAFS spectra of CoO@Pd at the Co K‐edge under ORR‐relevant potentials. Radial peaks B and C correspond to first‐shell Co─O and second‐shell Co─O/Co─Co scattering pathways, respectively. Relative to the as‐prepared state (Figure S14a), both peak B (including the B″ shoulder) and the overall EXAFS amplitude decrease substantially at V_oc_, indicating heavy OH^−^ adsorption (*OH^−^) on CoO_x_ (and metallic Co clusters, peak D), which converts the surface into a cobalt hydroxide–like phase. As the potential enters the ORR‐active region, peak B reaches its maximum intensity at 0.85 V, then drops sharply at 0.80 V, displaying clear splitting. At 0.70 V, the B″ feature disappears entirely, while peak C reaches its highest intensity. These trends reveal a progressive transformation of CoO_x_ into hydroxylated cobalt species during potential scanning. The strong *OH^−^ affinity of Co results in parallel 2e^−^ (H_2_O_2_) and 4e^−^ ORR pathways, suppressing overall catalytic activity. Corresponding Pd K‐edge EXAFS are shown in Figure 5c. Peaks E and F represent metallic Pd─Pd coordination and Pd‐*O^ads^ interactions, respectively, while region G (R > 3.5 Å) arises from multiple scattering of strongly bound ORR intermediates. Both E and F attain maximum intensity at 0.90 V, consistent with complete reduction of PdO surface species when the ORR rate is still low and *O^ads^ coverage remains minimal. At 0.80 V (the kinetic‐limiting regime), peak E weakens significantly, reflecting Pd lattice contraction caused by H incorporation from H_2_O reduction, coupled with disorder generated by accumulating *O^ads^. The reduction of the metallic shoulder E″ further confirms decreased near‐neighbor coherence typical for noble metals under strong adsorption. Region G becomes intense and broad at 0.80 V, indicating substantial buildup of stable intermediates beyond 3.5 Å from Pd centers, limiting ORR turnover. This is supported by the increase in peak F, evidencing excessive *O^ads^. At 0.70 V, peak F increases further, while peak E and E″ partially recover. The slight shift of peak E back toward the V_oc_ position reflects the electric‐field‐induced suppression of H diffusion into the Pd lattice, which weakens *O^ads^ binding and reduces intermediate accumulation—consistent with the observed decrease in region G intensity. Aforementioned scenarios reveal that Co is not an active ORR site in CoO@Pd. Its strong *OH^−^ affinity channels part of the reaction toward the 2e^−^ peroxide pathway. Pd is the primary O_2_ activation site, but the lack of synergistic interaction with neighboring Co or O_V_s leads to excessive accumulation of *O^ads^ and intermediates (peaks F and G), explaining the limited ORR activity. These conclusions agree with operando PFY‐XANES results (Figure 4(a)i–iii).

The Fourier‐transformed EXAFS spectra of CPCu‐1 at the Co K‐edge under ORR potential control are shown in Figure 5b. Radial peaks B and C correspond to backscattering contributions from Co─O and Co─O/Co─Co pairs in the first and second coordination shells, respectively. Compared with the as‐prepared state (Figure S14b), both peaks shift slightly toward lower R and exhibit markedly higher amplitudes at V_oc_, indicating that surface Co oxyhydroxide species undergo substantial coordination rearrangement. This increase in coordination number arises from the filling of O_V_s by *OH^−^ and *O^ads^, consistent with defect healing and the formation of a more hydroxylated Co environment. As the electrode potential is swept toward the diffusion‐limited region, the intensities of peaks B and C reach their maxima at 0.90 and 0.80 V, respectively, before decreasing again at 0.70 V. This sequence reflects the dynamic evolution of oxygenated intermediates on the Co sites. At 0.90 V, extensive accumulation of *O^ads^ saturates the Co‐centered vacancies in the first coordination shell, while the enhanced intensity of peak C suggests that additional *O^ads^ species populate more distal sites. When the potential decreases to 0.80 V (corresponding to the highest kinetic ORR current) rapid *O^ads^ turnover occurs due to the strong cooperative interaction between Co vacancies, neighboring Pd atoms, and surface hydroxide. This accelerates *O^ads^ consumption at the first shell (lower peak B amplitude) while promoting transient accumulation at the second shell (higher peak C amplitude), consistent with O‐intermediate migration away from Co prior to reduction. At 0.70 V, the higher reaction rate leads to renewed first‐shell *O^ads^ buildup, whereas second‐shell intensity decreases, indicating faster reduction and diminished residence of *O^ads^ in the outer coordination shells. The potential‐dependent Pd K‐edge FT‐EXAFS spectra of CPCu‐1 (Figure 5d) provide complementary insight. Peaks E and F arise from metallic Pd‐Pd coordination and Pd‐*O^ads^, respectively, while the region beyond R > 3.5 Å (region G) originates from multiple scattering involving adsorbed oxygenated intermediates. The positions of peaks E and F remain essentially unchanged across all potentials, demonstrating that *O^ads^ does not induce significant Pd surface oxidation or lattice reconstruction. At 0.80 V, peak E reaches its minimum intensity, whereas peak F reaches its maximum, signifying extensive adsorption of weakly bound *O^ads^ on the Pd surface and a slight reduction in local structural order. Upon entering the diffusion‐limited region, peak E recovers to its highest intensity while peak F remains nearly unchanged, confirming that rapid reduction at low potentials suppresses *O^ads^ coverage—an observation that strongly correlates with the Co K‐edge analysis. Region G exhibits negligible intensity changes throughout, further indicating that Pd predominantly mediates *O^ads^ transfer rather than acting as the primary site for O_2_ activation [31]. Integrating the operando EXAFS results with the PFY‐XANES analysis (Figure 4c‐i–iii) allows us to construct a coherent atomistic ORR mechanism for CPCu‐1. Oxygen vacancies around Cu (O_V_(Cu)) serve as high‐affinity O_2_ activation sites, splitting O_2_ into two *O^ads^ species. Vacancies surrounding Co provide strongly electronegative traps for incoming *O^ads^ and facilitate their subsequent reduction via reaction with H_2_O. Pd NPs, in contrast, function as O‐intermediate shuttles: they do not activate O_2_ directly but efficiently transfer surplus *O^ads^ generated at O_V_(Cu) sites toward available O_V_(Co) sites, thereby preventing local *O^ads^ oversaturation and sustaining fast ORR turnover [31]. This synergistic Cu‐Co‐Pd interaction establishes a highly efficient multi‐site catalytic cycle, wherein each element fulfills a distinct yet complementary role in O_2_ activation, intermediate migration, and final reduction.

**FIGURE 6:**
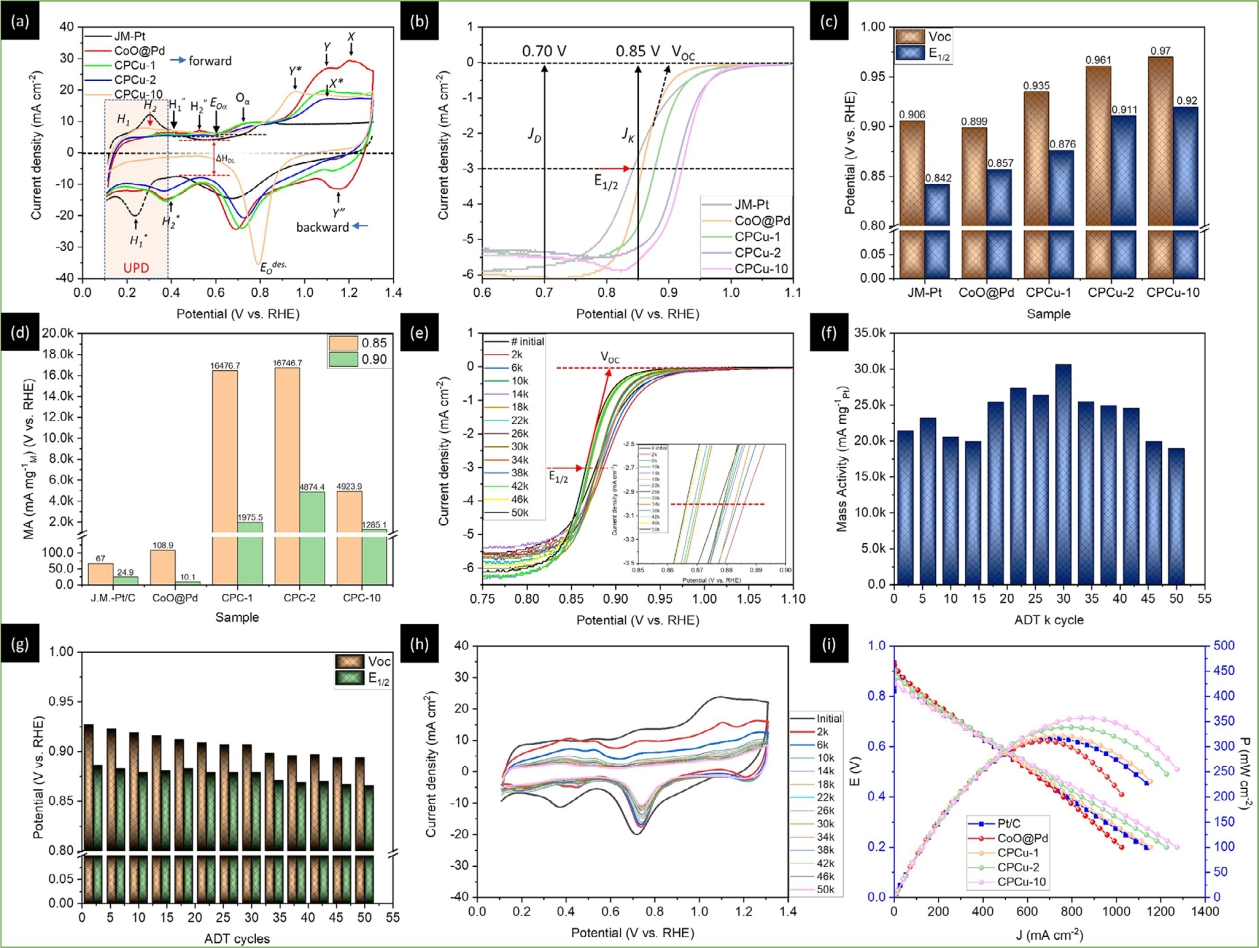
The (a) CV and (b) LSV curves of experimental, control, and standard (JM‐Pt) samples. (c) The V_oc_ and E_1/2_ values of samples in LSV analysis. (d) The mass activity (MA, mAmg_M_^−1^) of the evaluated samples at 0.85 and 0.90 V vs. RHE, where M represents Pt for JM‐Pt/C, Pd for CoO@Pd, and Cu for CPCu catalysts. (e) the LSV curves and corresponding (f) MA, (g) V_oc_ and E_1/2_, and (h) CV curves of CPCu‐1 at selected ADT cycles. (i) The polarization curves of samples in comparison in an alkaline fuel cell module.

Figure 6a compares the Cyclic voltammetry (CV) profiles of JM‐Pt, CoO@Pd, CPCu‐1, CPCu‐2, and CPCu‐10 catalysts were obtained to elucidate the evolution of surface redox and hydrogen adsorption behaviors under potential sweeping between 0.0–1.4 V (vs. RHE). In the **forward scan**, the characteristic peaks *H_1_* and *H_2_* correspond to hydrogen desorption from distinct Pd crystal facets, while *H_1_″* and *H_2_″* arise from potential shifts induced by interfacial strain and electronegativity gradients at the CoO_x_‐Pd heterojunctions (see magnified spectra in Figure S16). The onset potential *E_Oα_* denotes the formation of α‐phase surface oxides on Pd or Co‐rich sites, and the associated peak *Oα* represents the maximum oxide formation current. At higher potentials, peaks *Y* and *X* correspond to the oxidation of Co‐rich Pd and Pd‐rich Co domains, respectively. After Cu incorporation, new peaks *Y*⁎ and *X*⁎ emerge, attributed to the formation of mixed Pd─Co─Cu oxide species or local lattice reconstruction driven by atomic CuO_x_ decoration. During the **backward scan**, the reduction features *Y″* and *X″* reflect the reductive dissolution of surface oxides. The pronounced attenuation of these peaks upon Cu modification suggests a reduction in the number of electrochemically reducible surface sites. Notably, CPCu‐1 exhibits a distinct double‐layer (D_L_) charging region with exceptionally high current density, indicative of strong *OH^−^ adsorption affinity at the Cu‐decorated CoO_x_@Pd interface. This strong *OH^−^* binding stabilizes surface oxyhydroxides, thereby decreasing the degree of oxide reduction. Such a highly polarized interface facilitates *O^ads^ reduction kinetics, explaining the markedly enhanced oxygen reduction reaction (ORR) current density and MA observed for CPCu‐1. The potential *E_O_^des^* represents the reduction of *O^ads^ or surface oxides; its negative shift indicates that *O^ads^ reduction requires a smaller overpotential, consistent with the improved interfacial charge transport after Cu incorporation. The peaks *H_1_⁎* and *H_2_⁎* correspond to hydrogen adsorption on Pd surfaces, both shifting positively relative to CoO@Pd, reflecting stronger H binding and higher energy barriers for desorption induced by atomic CuO_x_ decoration. Compared with JM‐Pt, CoO@Pd shows significantly suppressed H desorption peaks due to the strong H─Pd interaction associated with high‐defect Pd nanocrystals, wherein atomic hydrogen is trapped in subsurface sites. The narrowing and intensification of the *O^des^* peak imply increased electroactive surface area and enhanced oxygen adsorption kinetics. Upon Cu modification (CPCu‐1), the H_2_ evolution feature becomes less pronounced and shifts toward higher potential, evidencing the increased H desorption barrier on CuO_x_‐modified Pd surfaces—an effect beneficial for promoting H_2_O dissociation during *O^ads^ reduction. The attenuation and slight potential shift of the high‐voltage *Y*/*X* oxidation peaks indicate that Cu atomic clusters reduce the number of oxidizable surface sites, a trend mirrored by the weakened *Y″* reduction feature in the reverse scan. When the Cu coverage increases further (CPCu‐2), both oxidation and reduction currents decrease slightly due to enhanced photoelectron and electrochemical shielding from thicker CuO_x_ overlayers, which partially block access to active Pd─Co interfacial sites. At the highest Cu loading (CPCu‐10, 10 wt.%), the current density in both the H_2_ evolution (UPD) and D_L_ regions markedly diminishes, signifying a substantial loss of H_2_ adsorption activity and weakened *OH^−^* affinity. The downward shift of the oxidation potential toward *Y⁎* implies a lower activation barrier for surface oxidation, whereas the disappearance of the backward high‐voltage reduction peak demonstrates the irreversibility of the Co─Pd dual‐atomic redox process at this stage. The narrowed and intensified *EO^des^* peak resembles that of pure Pd NPs, confirming that when CuO nanoclusters exceed 1–2 nm, the atomic‐scale Pd─Co─Cu synergistic coupling collapses, suppressing H adsorption and evolution on Pd sites. Overall, the CV results establish a clear correlation between Cu‐induced interfacial reconstruction, redox reversibility, and ORR kinetics. Atomic‐scale CuO_x_ decoration optimizes interfacial charge polarization and *OH^−^ affinity (CPCu‐1), whereas excessive Cu aggregation (CPCu‐10) introduces electronic shielding and disrupts Pd─Co synergy, diminishing catalytic efficiency.

Figure 6b compares the linear sweep voltammetry (LSV) curves of the catalysts during the ORR. Relative to commercial JM‐Pt, the CoO@Pd catalyst exhibits a higher diffusion‐limited current and a lower onset overpotential, indicating that electronic coupling between Pd and CoO_x_ significantly enhances O_2_ adsorption and activation. Upon introducing a small amount of Cu (CPCu‐1), the ORR polarization curve shows a pronounced current enhancement near 0.85 V (vs. RHE), along with a positive shift in the half‐wave potential (E_1/2_). This improvement reflects accelerated electron‐transfer kinetics and reduced redox overpotential, originating from atomic‐scale CuO_x_ decoration that induces charge redistribution at the Pd‐Co interface and stabilizes oxygenated intermediates. Figure 6c summarizes the open‐circuit potentials (V_OC_) and E_1/2_ of all samples. Accordingly, the Voc of CPCu‐1, CPCu‐2 and CPCu‐10 reach 0.935, 0.961, and 0.970 V (vs. RHE), respectively—both markedly higher than CoO@Pd (0.857 V) and JM‐Pt (0.842 V). These results indicate that, increasing the Cu loading to 10 wt.% (CPCu‐10) results in a highest Voc in LSV, implying that an excessively thick CuO_x_ overlayer facilitate the interfacial electronic communication and improving the surface reaction kinetics. It means that hetero‐structured CuO_x_ decoration significantly enhances interfacial electron transport efficiency and stabilizes surface oxygen intermediates, thereby promoting more favorable ORR kinetics. Figure 6d further compares the M_A_ at 0.85 and 0.90 V (vs. RHE). CPCu‐1 achieves the highest M_A_ of 16,478.7 mA mg_Cu_^−^^1^ and 10,975.5 mA mg_Cu_^−^^1^, outperforming JM‐Pt (67 mA mg_Pt_^−^^1^) and CoO@Pd (108.9 mA mg_Pd_^−^^1^) by more than two orders of magnitude. When the Cu loading increases to 10 wt.%, the MA decreases drastically to 4,923.9 mA mg_Cu_^−^^1^, confirming that excessive Cu results in electron localization, thickening of surface oxide layers, and reduced availability of active sites. Taken together, these results reveal the following structure–activity relationships:

- Atomic‐scale CuO_x_ decoration (CPCu‐1) modulates the Pd─Co electronic structure, optimizes *O^ads^ binding energy, and maximizes ORR kinetics and MA.
- Moderate Cu coverage (CPCu‐2) maintains efficient charge transport and introduces beneficial interfacial strain, retaining high activity.
- Over‐decorated CuO_x_ shells (CPCu‐10) hinder electron coupling and impose a surface‐blocking effect, resulting in decreased ORR performance.

Overall, this study identifies an “atomic‐to‐subnanometer CuO_x_ interface reconstruction–mediated enhancement mechanism” in CPCu‐1, providing both experimental and mechanistic validation for designing highly active Pd─Co─Cu multi‐metal ORR catalysts. Based on operando EXAFS, PFY‐XANES, and electrochemical kinetics, the ORR mechanism is proposed as a O_V_(**Cu)‐initiated O_2_ activation → O_V_(Co)‐O^ads^ reduction → Pd‐mediated O^ads^ transfer** sequence:

**O_2_ Activation at O_V_(Cu)**

1. O_V_(Cu) + O_2(g)_ **→** O_V_(Cu)‐O_2_^ads^ (rapid)
2. O_V_(Cu)‐O_2_^ads^ + O_V_(Cu) **→** 2 O_V_(Cu)‐O^ads^ (rapid dissociation)

**O^ads^ Migration to O_V_(Co) (Spillover Pathway)**

3. O_V_(Cu)‐O^ads^ + O_V_(Co) → O_V_(Cu) + O_V_(Co)‐O^ads^ (Cu vacancy regeneration; O^ads^ relocation)

**Rate‐Determining O─O Bond Reduction at O_V_(Co)**

4. 2 O_V_(Co)‐O^ads^ + 2 H_2_O + 4 e^−^ → 4 OH^−^

(O^ads^ reduction; slow / RDS)

In the subsequent reduction stage, the Co‐associated oxygen vacancies serve as the primary reduction centers for the migrated O^ads^ species. Rather than invoking a free H^ads^ intermediate, the transformation of O^ads^ into OH^ads^/OH^−^ is more reasonably described as a water‐assisted electron‐transfer process under alkaline ORR conditions. In this process, H_2_O acts as the proton source, while the external circuit supplies electrons to progressively reduce the adsorbed oxygen species. Therefore, the rate‐determining reduction step can be expressed as:

$$O_{V}\left(\mathrm{Co}\right)-O^{\mathrm{ads}}+H_{2}O+e^{-}\to O_{V}\left(\mathrm{Co}\right)-OH^{\mathrm{ads}}+OH^{-}$$

followed by:

$$O_{V}\left(\mathrm{Co}\right)-OH^{\mathrm{ads}}+e^{-}\to O_{V}\left(\mathrm{Co}\right)+OH^{-}$$

This pathway simultaneously accounts for OH^−^ generation and regeneration of the Co‐associated oxygen‐vacancy site.

**Pd‐Mediated O^ads^ Transfer and Flux Control**

5. Pd‐O^ads^ (spillover from step 2) + Pd(F) → Pd(F)‐O^ads^ → Pd + O_V_(Co)‐O^ads^:

(Pd does not activate O_2_; it redistributes O^ads^ to Co sites and prevents local oversaturation)

This multi‐site ORR pathway illustrates a cooperative division of labor:

➢ **O_V_s in CuO_x_**: drive ultrafast O_2_ activation
➢ **O_V_s in CoO_x_**: serve as strong O^ads^ traps and primary reduction centers
➢ **Pd NPs**: mediate lateral O^ads^ diffusion (spillover), preventing bottleneck accumulation

Such synergy explains the exceptional ORR activity of CPCu‐1 (steps 1 – 5), directly correlating with their maximized E_1/2_, *J*_K_, and MA. For the case of CPCu‐10, O_2_ activation occurs at Pd atoms (step 6). After that the O^ads^ migrate to the neighboring CoO_x_ sites (step 7) and then interacting with water molecules (step 4) to completing the ORR.

6. Pd + O_2(g)_ → Pd‐O_2_^ads^ + Pd → 2 Pd‐O^ads^
7. 2 Pd‐O^ads^ + 2 O_V_(Co) **→** 2 Pd + 2 O_V_(Co)‐O^ads^

**SCHEME 1:**
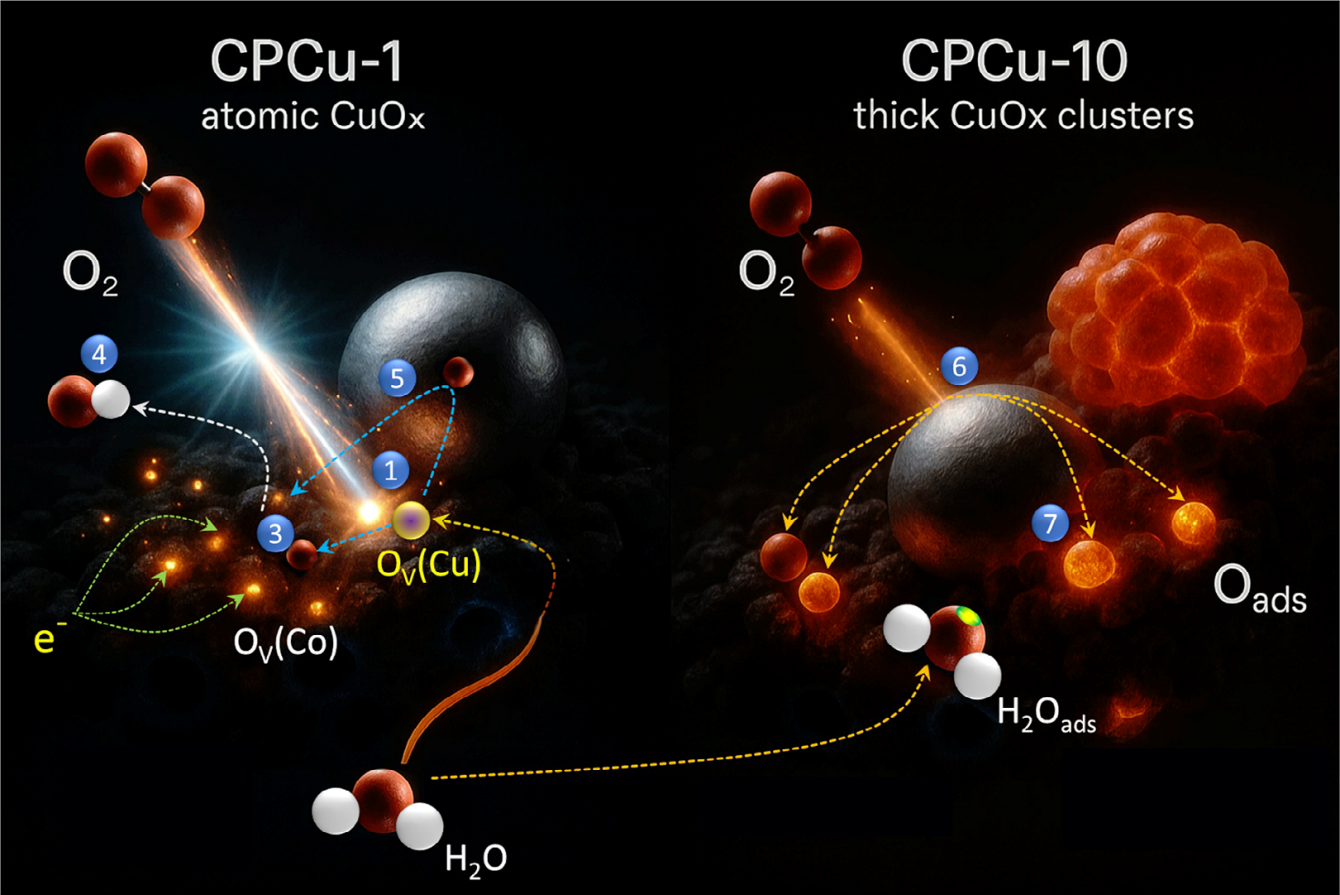
Schematic representation for the reaction pathways of CPCu‐1 and CPCu‐10 in ORR.

A schematic representation for the reaction pathways of CPCu‐1 and CPCu‐10 is shown in Scheme 1.

**FIGURE 7:**
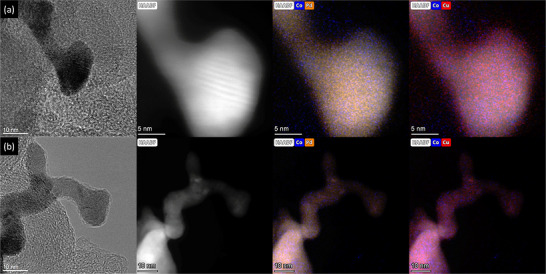
HRTEM, HADDF‐STEM, and corresponding elemental EDS maps of Co‐Pd and Co‐Cu for the post ADT (a) CPCu‐1 and (b) CPCu‐10.

The durability evolution of the CPCu‐1 catalyst during the accelerated degradation test (ADT, 1st – 50 000th cycles) was systematically analyzed by combining LSV, MA, CV, and potential tracking data. The LSV curves (Figure 6e) reveal a gradual decrease in diffusion‐limited current density with increasing cycle number, accompanied by a slight positive shift in both the V_OC_ and E_1/2_ up to 30 k cycles, followed by a decline toward the initial values after 50 k cycles. This transient enhancement indicates an early‐stage structural relaxation and oxygen‐vacancy redistribution that temporarily improve interfacial conductivity and O_2_ adsorption kinetics. The MA (Figure 6f) exhibits the same trend, reaching a maximum of approximately 3.0 × 10^4^ mA mg_Cu_^−^^1^ at 30 k cycles before gradually decreasing, suggesting that the dissolution of CuO_x_ and CoO_x_ layers first exposes new active sites and then weakens the Pd─O─Cu synergetic interface. The potential parameters (Figure 6g) confirm this evolution, with V_OC_ and E_1/2_ peaking near 30 k cycles, consistent with the operando PFY‐XANES/EXAFS results showing a temporary stabilization of the Co^3^^+^/Co^2^^+^ ratio and Pd─O─Cu coordination before gradual degradation of the oxide shell. Meanwhile, the CV profiles (Figure 6h) display a progressive attenuation of the double‐layer current and transformation toward the characteristic pattern of metallic Pd, indicating that the highly hydrophilic CoO_x_ species are progressively leached out, removing the inter‐particle diffusion barrier and facilitating Pd NPs coalescence—a process corroborated by post‐ADT HRTEM images (Figure 7). For easy calcification, Figure 6h and the its double layer region were presented in Figure S15. These observations collectively suggest a three‐stage degradation mechanism: (i) interfacial self‐reconstruction and electronic redistribution during 0–10 k cycles, (ii) maximized Pd─O─Cu cooperative activity between 10 k and 30 k cycles, and (iii) interfacial collapse and Pd agglomeration beyond 30 k cycles. Thus, the transient enhancement and subsequent decline of ORR performance originate from the dynamic restructuring of the CuO_x_‐CoO_x_ heterointerface, demonstrating that the long‐term catalytic stability of multi‐metallic systems is ultimately governed by the persistence of electronic coupling and defect‐mediated synergy at the oxide–metal boundary.

Figure 6i presents the polarization (E–J) and power density (P–J) curves of alkaline fuel cells (AFCs) employing Pt/C, CoO@Pd, CPCu‐1, CPCu‐2, and CPCu‐10 as cathode catalysts. Details for the AFC test were given in the supplementary information. Under identical operating conditions, all samples exhibit the three characteristic regions of fuel cell operation: the activation‐controlled region at low current density, the ohmic region at intermediate current density, and the mass‐transport‐limited region at high current density. Commercial Pt/C delivers an open‐circuit voltage (E_0_) of approximately 0.92 V and a peak power density of **∼270 mW cm^−^^2^**, serving as a benchmark. The CoO@Pd catalyst shows lower overall performance, with a maximum power density of **∼230 mW cm^−^^2^**, indicating that although CoO_x_‐Pd heterointerfaces enhance the ORR onset potential, the system remains constrained by Pd surface oxidation and suboptimal electron‐transfer kinetics. CPCu‐1 exhibits the highest AFC performance, achieving a maximum power density of **∼430 mW cm^−^^2^**, surpassing Pt/C by nearly **60%**. Its polarization curve features a notably slower voltage decay across the intermediate and high‐current regions, reflecting significantly improved electronic conductivity and enhanced oxygen‐reduction kinetics under practical cell operation. This behavior correlates well with the high E_1_/_2_ and MA observed in RDE measurements. The enhancement originates from the atomic‐scale CuO_x_ layer, which (i) exhibits strong O_2_ dissociation activity at O_V_(Cu), (ii) promotes efficient electronic injection at the Pd‐Co interface, (iii) enables O_V_(Co) to serve as strong O^ads^ trapping sites, and (iv) leverages Pd atoms to redistribute excess *O^ads^—generated at O_V_(Cu)—toward more distant O_V_(Co)s for subsequent reduction. This division of labor minimizes local *O^ads^ accumulation and maintains rapid multielectron transfer. CPCu‐2 delivers a slightly lower peak power density (**∼390 mW cm^−^^2^**) but still significantly outperforms Pt/C, demonstrating that although a moderately thicker Cu layer introduces minor shielding effects, the interfacial synergistic reactions remain largely preserved. In contrast, CPCu‐10 reaches only **∼360 mW cm^−^^2^**, and its E–J curve shows steep voltage decay at high current density, indicating severe mass‐transport and electron‐transfer limitations. These trends align with earlier CV/LSV results, where excessive CuO_x_ formed a nonconformal coating that hindered oxygen diffusion and restricted active‐site accessibility. Overall, the CPCu catalyst series reveals that introducing an optimally thin, atomic‐scale CuO_x_ overlayer onto Pd‐CoO_x_ heterostructures enhances electronic conductivity, accelerates O_2_ reduction, and improves water‐formation coupling during ORR. Among these, CPCu‐1 provides the most favorable interfacial structure, enabling high power output and excellent electrochemical stability under fuel‐cell operating conditions. These results underscore the effectiveness of hierarchical Pd‐Co‐Cu interfaces in lowering kinetic barriers and sustaining rapid four‐electron ORR pathways in alkaline membrane systems.

In summary, we demonstrate that atomic‐scale CuO_x_ interface reconstruction at CoO_x_‐supported Pd NPs provides a powerful lever to control the electronic structure, defect chemistry, and reaction cooperativity of multi‐metal ORR catalysts. Structural analyses reveal that low Cu loadings generate ultrathin, conformal CuO_x_ layers with atomically dispersed Cu‐O‐M motifs and high oxygen‐vacancy density, whereas higher loadings drive the formation of nanometer‐scale CuO_x_ islands that partially decouple Pd‐Co interfaces and introduce electronic shielding. XANES/EXAFS and XPS measurements show that the optimally decorated catalyst (CPCu‐1) stabilizes metallic Pd, maintains oxygen‐deficient Co^3^^+^ environments, and hosts electronically flexible Cu centers that collectively promote interfacial charge polarization and tunable *O^ads^ binding. Operando PFY‐XANES/EXAFS under ORR conditions resolve a CuO_x_‐initiated O_2_ activation → Co‐vacancy‐mediated *O^ads^ reduction → Pd‐assisted *O^ads^ redistribution sequence, in which Cu vacancies act as high‐affinity O_2_ splitting sites, O_V_(Co)s provide strongly coupled *O^ads^ reduction centers, and Pd NPs function as metallic shuttles that balance O‐intermediate flux and suppress local poisoning. This tri‐metallic division of labor maximizes ORR kinetics, yielding E_1/2_ up to 0.935 V vs. RHE and MAs approaching ∼1.6 × 10^4^ mA mg_Cu_^−^^1^, more than two orders of magnitude higher than commercial Pt/C. When integrated into alkaline fuel cells, CPCu‐1 delivers peak power densities of ∼430 mW cm^−^^2^, surpassing Pt/C by nearly 60%, and exhibits a characteristic three‐stage durability evolution governed by dynamic CuO_x_‐CoO_x_ interface self‐reconstruction. To further validate the enhancement in overall catalytic performance induced by Cu modification, and to eliminate potential ambiguity associated with normalization criteria, we recalculated the mass activity using Pd as the reference active component. The corresponding results are summarized in Table S3. Upon incorporation of Cu species, and normalizing based on the combined mass of Pd and Cu as active components, the mass activities at 0.85 V vs. RHE are determined to be 242.3 mA mg^−^^1^_(Pd+Cu)_ for CPCu‐1, 485.5 mA mg^−^^1^_(Pd+Cu)_ for CPCu‐2, and 748.1 mA mg^−^^1^_(Pd+Cu)_ for CPCu‐10. Compared to the pristine CoO@Pd catalyst (108.9 mA mg^−^^1^Pd), the introduction of Cu results in an enhancement of Pd‐based mass activity by factors of 1.22, 3.46, and 5.87 for CPCu‐1, CPCu‐2, and CPCu‐10, respectively. This trend is in excellent agreement with the evolution of kinetic current density (Jk), collectively demonstrating that Cu modification plays a decisive role in enhancing reaction kinetics and overall catalytic efficiency in the ternary system. These findings establish Pd‐Co‐Cu heterointerfaces with atomic‐scale CuO_x_ reconstruction as an effective platform for reconciling high activity, reduced noble‐metal usage, and robust durability in alkaline ORR catalysts (see Table S4 for the benchmark of Cu based catalysts in literature). More broadly, the mechanistic insights obtained here—linking vacancy‐engineered O_2_ activation, metal–oxide charge coupling, and operando *O^ads^ flux control—offer design principles that can be extended to a broad range of multi‐site electrocatalytic reactions in fuel cells and beyond.

## 4 Conclusion

In this work, we demonstrate that atomic‐scale CuO_x_ interface reconstruction on CoO_x_‐supported Pd NPs provides an effective handle to engineer the electronic structure, defect chemistry, and reaction cooperativity of multi‐metal ORR catalysts. Systematic electron microscopy and XRD analyses reveal a clear structural evolution with increasing Cu loading: at low Cu content (**CPCu‐1**), ultrathin and conformal CuO_x_ layers with atomically dispersed Cu─O─M (M = Pd, Co) motifs are formed, which suppress Pd coalescence, introduce abundant surface defects, and maximize oxygen‐vacancy density. At higher loadings (CPCu‐10), these species transform into 1–2 nm CuO_x_ islands that partially decouple the Pd‐Co interface, increase structural ordering around Pd, and generate an electronically shielding overlayer that limits interfacial communication and mass transport. XANES/EXAFS and XPS measurements corroborate this structure evolution and show that the optimally decorated catalyst, CPCu‐1, stabilizes metallic Pd, preserves oxygen‐deficient Co^3^^+^ environments, and introduces electronically flexible Cu centers and Cu‐derived O_V_s at the outer surface. These features collectively promote interfacial charge polarization, tune the Co and Cu 4s/4p band structures, and provide a balanced *O^ads^ binding strength across the Pd─Co─Cu heterointerface. Operando PFY‐XANES and EXAFS under ORR‐relevant conditions resolve a multi‐site reaction sequence in which Cu vacancies at CuO_x_ domains function as high‐affinity O_2_ splitting centers, Co vacancies act as strongly coupled *O^ads^ reduction sites, and Pd NPs serve mainly as metallic shuttles that mediate lateral *O^ads^ transfer and regulate O‐intermediate flux. This tri‐metallic division of labor suppresses local intermediate accumulation, minimizes peroxide pathways, and maximizes four‐electron ORR kinetics. Electrochemical characterization confirms that this atomic‐scale interface design translates directly into outstanding catalytic performance. CPCu‐1 exhibits a E_1/2_ of 0.876 V vs. RHE and a MA approaching 1.6 × 10^4^ mA mg_Cu_^−^^1^ (without decay in an acceleration degradation test for 50 000 cycles), exceeding commercial Pt/C and CoO@Pd by more than two orders of magnitude. Alkaline fuel cell tests further demonstrate that CPCu‐1 delivers a peak power density of ∼430 mW cm^−^^2^, nearly 60% higher than Pt/C, and displays a characteristic three‐stage durability evolution governed by dynamic CuO_x_‐CoO_x_ self‐reconstruction at the oxide–metal interface. The transient performance enhancement followed by gradual decline highlights both the potential and the intrinsic limits of defect‐rich multi‐metal interfaces, emphasizing that long‐term stability is ultimately controlled by the persistence of electronic coupling and vacancy‐mediated synergy. Overall, this study establishes hierarchical Pd─Co─Cu heterointerfaces with atomic‐to‐sub‐nanometer CuO_x_ reconstruction as a powerful platform for reconciling high ORR activity, reduced noble‐metal usage, and robust fuel‐cell performance in alkaline media. More broadly, the mechanistic insights obtained here—linking vacancy‐engineered O_2_ activation, metal–oxide charge coupling, and operando O^ads^ flux control—provide transferable design principles for constructing next‐generation multi‐site electrocatalysts for oxygen‐related reactions and other complex multi‐electron processes in electrochemical energy‐conversion systems.

## Author Contributions

**Ching‐Hua Fan**: data curation. **Ting‐Shan Chan**: methodology, data curation. **Po‐Chun Chen**: methodology, supervision, funding acquisition. **Yang‐Yang Hsu**: data curation. **Nozomu Hiraoka**: data curation, methodology. **Kuan‐Wen Wang**: methodology. **Hirofumi Ishii**: methodology, data curation. **Tsan‐Yao Chen**: funding acquisition, investigation, conceptualization, methodology, validation, visualization, project administration, formal analysis, supervision, resources, writing – original draft, writing – review and editing. **Kuang‐Kuo Wang**: methodology, data curation.

## Conflicts of Interest

The authors declare no conflicts of interest.

## Supporting information




**Supporting File**: advs76006‐sup‐0001‐SuppMat.docx.

## Data Availability

The data that support the findings of this study are available from the corresponding author upon reasonable request.
