# Supplementary material for: Atomic‐Level CuOx‐CoOx‐Pd Interfacial Engineering Enables Hierarchical Synergy for High‐Efficiency ORR Pathways and Boosted Power Output in Alkaline Fuel Cells
Source: Adv Sci (Weinh). 2026 Jun 4:e76006. Online ahead of print. doi: 10.1002/advs.76006 (PMC13336373; doi:10.1002/advs.76006)
Supplement: Supplementary file 1 — Supporting File: advs76006‐sup‐0001‐SuppMat.docx. [file ADVS-9999-e76006-s001.docx]

**Supporting Information for**

**Atomic-Level CuO_x_-CoO_x_-Pd Interfacial Engineering Enables Hierarchical Synergy for High-Efficiency ORR Pathways and Boosted Power Output in Alkaline Fuel Cells**

Yang-Yang Hsu,^a^ Ching-Hua Fan,^b^ Kuan-Wen Wang,^c^ Kuang-Kuo Wang,^d^ Nozomu Hiraoka,^e^ Hirofumi Ishii,^e^ Ting-Shan Chan,^e^ Po-Chun Chen,^b*^ and Tsan-Yao Chen^a,g,h,i*^

^a.^ Department of Engineering and System Science, National Tsing Hua University, Hsinchu 30013, Taiwan

^b.^ Department of Materials and Mineral Resources Engineering, National Taipei University of Technology, Taipei 10608, Taiwan.

^c.^ Institute of Materials Science and Engineering, National Central University, Taoyuan City 32001, Taiwan

^d.^ Department of Materials and Optoelectronic, National Sun Yat-sen University

^e.^ National Synchrotron Radiation Research Center, Hsinchu 30076, Taiwan

^f.^ Institute of Analytical and Environmental Science, National Tsing Hua University, Hsinchu 30013, Taiwan

^g.^ Institute of Nuclear Engineering and Science, National Tsing Hua University, Hsinchu 30013, Taiwan

^h.^ Department of Chemical Engineering, Chung-Yuan Christian University, Taoyuan City 32314, Taiwan

1. **Sample preparation**

The hierarchically structured Co oxide (CoO_x_) supported Pd NPs with Pt trimer decoration (CPP) catalysts were synthesized by self-aligned wet chemical method with sequence and reaction time controls for the heterogeneous nucleation and crystal growth of the three elements (**Scheme S1**). Prior to the crystal growth, the catalyst support (i.e. Active carbon, XC-72) was functionalized with acid treatment by microwave annealing for strengthening the attachment of catalysts on its surface. After the acid treatment with 4.0 M H_2_SO_4_ solution in microwave oven at a power of 2000W for 30 mins, the powder was washed by double-distilled water until the pH value of rinsing water is 6.0. In the **step-1**, 1.2 g of 5.0 wt% XC-72 solution in D.I. water (i.e., containing 60 mg of XC-72 powder) is dispersed in an aqueous solution containing 72.7 mg CoCl₂·6H₂O (CoCl₂·6H₂O, 99%, Sigma-Aldrich Co.) and stirred at 200 rpm for 2 h. The as-prepared mixture contains 0.306 mmoles (18 mg) of Co^2+^ ions adsorbed on XC-72 with a weight ratio of 30 wt.% for Co/AC (namely Co^2+_ads^-AC_(aq)_). In **step-2**, a 5.0 ml of D.I. water solution comprising 0.11 g of sodium borohydride (NaBH_4_; 99%, Sigma-Aldrich Co.) was instantly dropped into the Co^2+_ads^-AC_(aq)_ and stirred at 200 rpm for 10 seconds. In this step, metastable Co metal nanoparticles were formed (i.e. Co-AC), which later turned into Co-oxide on XC-72 in the solution (i.e. CoO_x_-AC_(aq)_) by interacting with the O_2_ molecules (O_2(aq)_) in **step 3**. Subsequently, 3.05 g of Pd precursor solution (1.0 M HCl_(aq)_ containing 54.2 mg of PdCl_2_.) was added into CoO_x_-AC_(aq)_ to grow the Pd-NPs on Co/CoO_x_ support (namely Co@Pd). In this step (**step 4**), Pd^2+^ ions are reduced by the excess NaBH_4_ in the **step-2** and deposited on the Co/CoO_x_ surface. The Pd precursor solution was prepared by dissolving palladium chloride (PdCl_2_, 99%, Sigma-Aldrich Co.) in 1.0 M of HCl_(aq)_. In Co@Pd, the molar ratio is 1.0 for Pd/Co. After the synthesis of Co@Pd, an appropriate amount of Pt-precursor was added into the solution of Co@Pd (**step 5**). The as-prepared Co@Pd NC were pre-treated by ultrasonication for creating sub-nano defects prior to the injection of the Cu precursor. These defects anchor the Cu species and then control the dimension of the Pt decoration. The Cu²⁺ precursor solution (78.7 mM Cu²⁺) was prepared by dispersing 259.1 mg of CuCl_2_·2H_2_O (corresponding to 1.520 mmol of Cu²⁺) in 19.31 g of deionized water under magnetic stirring at room temperature until complete dissolution. For controlling the size and distribution of Cu-decoration, the loading of Cu is properly adjusted for controlling the dimension of Cu species in atomic trimer, sub-nano cluster and nanoparticle. In this article, the catalysts are denoted as CPCu-1, CPCu-2 and CPCu-10 for that containing 1.0, 2.0 and 10.0 wt.% of Cu atoms, respectively.

1. **Physical Characterizations**

The atomic compositions of the CPCu catalysts were determined by the inductively coupled plasma-atomic emission spectrometer (ICP-AES, Jarrell-Ash ICAP 9000) and results are shown in **Table S1**. **The physical structures were determined by cross-referencing results of the microscopy and X-ray spectroscopy inspections.** Aberration-corrected STEM characterization was performed on a Thermo-Fisher Themis Z microscope equipped with two aberration correctors under 300 kV. High angle annular dark-field (HAADF)-STEM images were recorded using a convergence semi angle of 11 mrad, and inner- and outer collection angles of 59 and 200 mrad, respectively. Energy-dispersive X-ray spectroscopy (EDS) was carried out using 4 in-column Super-X detectors. X-ray powder diffraction (XRPD) with synchrotron X-ray sources was employed for determining the crystal structure and corresponding patterns were collected at the beamline of BL-01C2 of National Synchrotron Radiation Research Center (NSRRC), Taiwan with the incident X-ray of wavelength 0.7749 Å (16 KeV). The X-ray absorption spectroscopy (XAS) was applied to unveil the local chemical structure. The typical XAS spectra of CPP catalysts at Cu K-edge, Co K-edge and Pd K-edge were measured in fluorescence mode at beamlines BL-17C and 01C1 of NSRRC, Taiwan. X-ray photoelectron spectroscopy (XPS), carried out at beamline BL-24A1 of NSRRC, Taiwan, was used to investigate the oxidation states and surface compositions. **The proposed ORR pathways were** confirmed by cross-referencing the results of the *in-situ* partial fluorescence yield (PFY)-XAS inspections. The in-situ PFY-XAS inspection at Cu K-edge, Pd K-edge and Co K-edge was performed at the beamline of BL-12XU with a customized single-compartment Teflon-made electrochemical cell at Spring-8, Japan. The samples for PFY-XAS analysis were prepared by dropping 300 micro-liter of catalyst slurry on the conducting graphite slit. The catalyst slurry was prepared with the same recipe as that of electrochemical measurements (*more details are given in the subsequent section*). During the measurement, the sample (i.e., catalyst-coated graphite plate) is placed in an electrochemical cell filled with oxygen-saturated electrolyte (0.1 M KOH). The sample side is faced with the incident X-ray where the window is sealed by a 10 μm thick Kapton tap.

**Table S1** Inductively Coupled Plasma (ICP) Mass Spectroscopy determined elementary concentrations of CPCu-1, CPCu-2 and CPCu-10

| Sample | Pd (wt%) | Co (wt%) | Cu (wt%) | C (wt%) |
| --- | --- | --- | --- | --- |
| CPCu-1 | 25.73 | 10.61 | 0.288 | 63.37 |
| CPCu-2 | 25.00 | 10.05 | 0.597 | 64.35 |
| CPCu-10 | 24.01 | 9.894 | 3.150 | 62.94 |


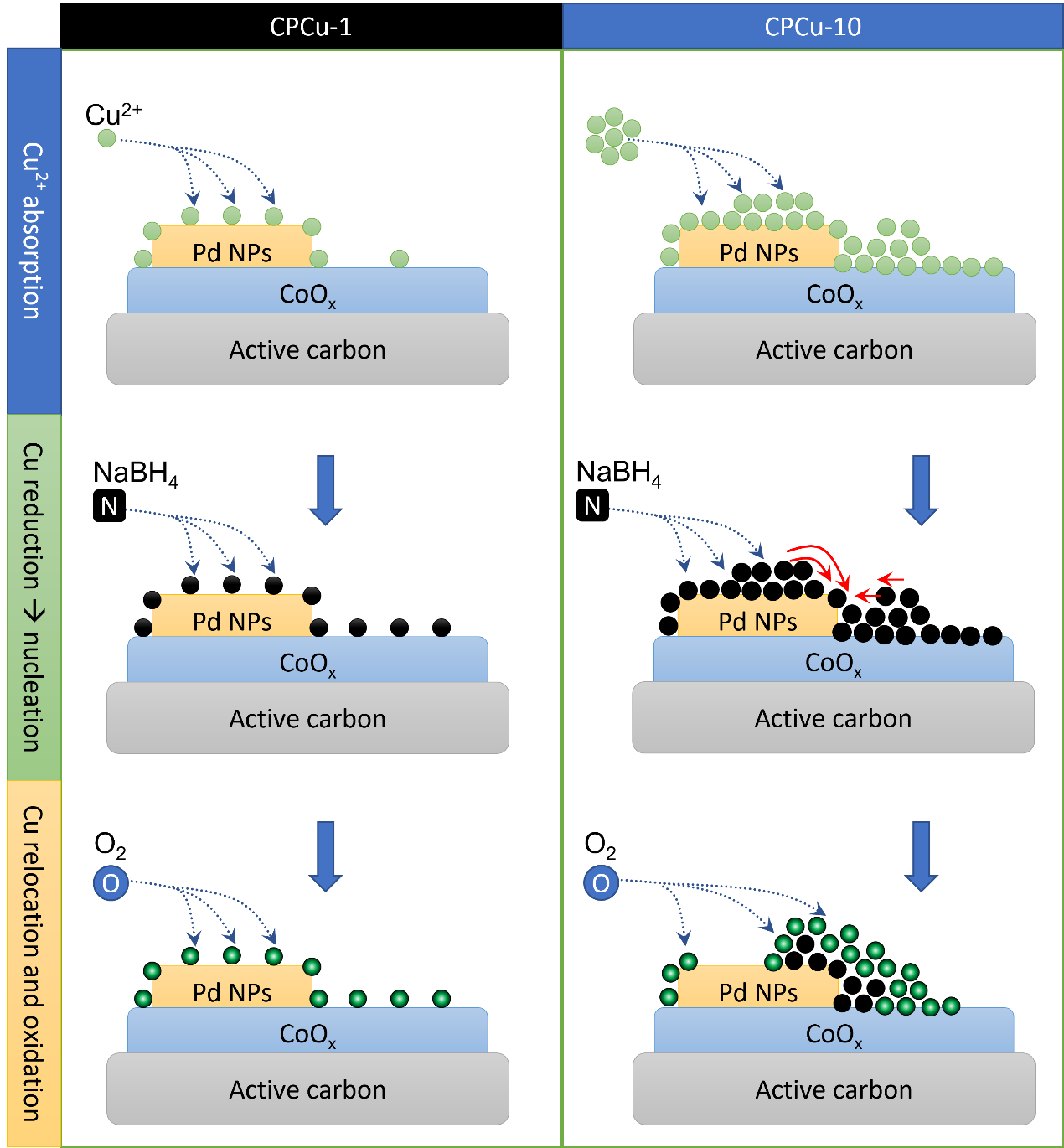


**Scheme S1** Schematic representation for the crystal growth of CPCu-1 and CPCu-10.

1. **Electrochemical Analysis method.**

An electrochemical cell (CH Instruments Model 600B, CHI 600B) equipped with a three-electrode system was used for all electrochemical measurements. The catalyst slurry for the ORR experiment was prepared by dispersing 5.0 mg of catalyst powder in 1.0 ml of isopropanol (IPA) containing 50 μl of conducting binder (Nafion-117, 99%, Sigma-Aldrich Co.). The mixture was subjected to ultrasonication for 30 minutes prior to the ORR test. For ORR measurements, 10.0 μl of catalyst slurry was drop cast and air-dried on a glassy carbon rotating disk electrode (RDE) (0.196 cm^2^ area) as the working electrode. Hg/HgCl_2_ (the voltage was calibrated by 0.242 V, in alignment with that of RHE) electrode saturated in KCl aqueous solution was used as a reference electrode. Whereas, a graphite rod was employed as the counter electrode to avoid the Pt contamination. The cyclic voltammetry (CV) was obtained in the in KClO_4_ solution (0.1 M) in the potential range of 0.1 V to 1.3 V (V vs RHE.) at the voltage scan rate of 0.05 V s^−1^. The linear sweep voltammetry (LSV) was conducted in an aqueous alkaline electrolyte solution of 0.1 M KOH (pH = 13) in the potential range from 0.4 V to 1.1 V (V vs RHE.) at the voltage scan rate of 0.001 V s^−1^. The rotation rate of 400-3600 rpm was used for LSV. N_2_ and O_2_ atmosphere were used for CV and LSV, respectively. The durability of the experimental NCs was evaluated using an accelerated degradation test (ADT) in the potential range of 0.5V to 1.0V (V vs RHE.) with the applied scan rate of 0.05 Vs^-1^ in O_2_ atmosphere. All the electrochemical experiments were performed at room temperature. The detailed procedure for the ORR mass activity calculation has been given in the following section.

For CO-stripping analysis, the adsorption of CO on the surface of the catalyst was performed initially by purging CO into 0.5 M H_2_SO_4_ at 0.05 V (vs RHE) for 20 min. Subsequently, the CO stripping voltammetry was measured between -0.10 and 1.20 V (vs RHE) in N_2_ saturated 0.5 M H_2_SO_4_ solution at a scan rate of 50 mVs^-1^. A glassy carbon electrode, Pt wire and Ag/AgCl electrode were used as the working, counter and reference electrodes, respectively.

1. HAADF-STEM analysis on CoO@Pd-Cu





**Figure S1** line scan histogram for the EDS maps of Figure 1(b) and 1(c).

1. X-rat Powder Diffraction analysis results

**Figure S2a** presents the X-ray diffraction (XRD) patterns of the experimental catalysts (CPCu-1, CPCu-2, and CPCu-10), the control sample (CoO@Pd), and the reference Pd-AC catalyst. Diffraction peaks labeled A-E correspond to the (111), (200), (220), (311), and (222) reflections of FCC Pd metal, whereas peaks **F** and **G** arise from the (211) facet of Co_3_O_4_.[27] The emergence of peak G confirms that Pd and Co atoms partially dissolve into each other during synthesis to form a Co_2_Pd solid-solution alloy **(**the (10-13) facet**)**. Meanwhile, the appearance of Co_3_O_4_ reflections results from the oxidation of exposed Co species upon air exposure, further establishing that all samples consist of Pd NPs supported on cobalt oxides. The Pd-related diffraction peak positions remain unchanged across all Cu-modified samples and closely match those of CoO@Pd, indicating that Cu does not diffuse into the Pd lattice to form a Pd-Cu solid solution under the present synthetic conditions. Instead, the progressive decrease in diffraction intensity with increasing Cu loading suggests that the crystallinity of Pd NPs decreases as the surface CuO_x_ layer becomes thicker and introduces additional structural disorder.[1] **Figure S2b** summarizes the coherent domain size (D_{hkl}_) calculated from the Scherrer equation for each Pd lattice plane. The obtained D_{hkl}_ values are universally much smaller than the particle diameters observed by HRTEM (D_{avg}_), reflecting the presence of substantial surface defects and lattice strain, which significantly reduce the size of the periodically ordered atomic regions inside the NPs. Compared with the CoO@Pd control, all Cu-decorated samples exhibit smaller D_{hkl}_, confirming that Cu-induced surface oxidation increases the degree of structural disorder on Pd surfaces. The variations in ∆D(Pd) relative to CoO@Pd are shown in **Figure S2c**. With the exception of the (200) plane, CPCu-1 exhibits the smallest D_{hkl}_, indicating that 1 wt% Cu decoration creates the highest defect density on Pd NPs. At higher Cu loadings, D_{hkl}_ gradually increases, suggesting that the thicker CuO_x_ overlayer provides a steric protection effect, which inhibits excessive oxidation or structural roughening at Pd-Pd interparticle contact regions. The influence of Cu decoration on the Co_3_O_4_ and Co_2_Pd phases is further revealed in **Figure S2d**.[34] The D_{hkl}_ of Co_3_O_4_ decreases with increasing Cu loading up to 2 wt%, consistent with the aforementioned steric protection effect that suppresses oxidative restructuring around Co sites (i.e., formation of defect sites). In parallel, the growth of the Co₂Pd alloy phase becomes more pronounced, indicating that moderate Cu decoration helps stabilize the metallic interface and facilitates alloy formation. When the Cu content reaches 10 wt%, however, the D_{hkl}_ of Co_3_O_4_ increases. This reversal suggests that high Cu loading leads to heterogeneous nucleation of CuO_x_ domains on Pd surfaces, reducing the extent of Cu coverage on Co-containing regions. As a result, the Co-Pd interface becomes more exposed, slightly enhancing Co-Pd alloy formation. A schematic illustration of the structural evolution as a function of Cu content is provided in **Scheme S1**, highlighting the interplay between Cu-induced surface disorder, steric protection, alloy formation, and heterogeneous CuO_x_ nucleation pathways.





**Figure S2** (a) X-ray diffraction (XRD) patterns of the synthesized catalysts, including CPCu-1, CPCu-2, and CPCu-10, together with the control sample (CoO@Pd) and reference catalyst (Pd-AC). (b) Coherent domain sizes of the face-centered cubic (FCC) crystallographic facets (D_{hkl}_) for the experimental samples, as estimated using the Scherrer equation. (c) Relative percentage deviation in coherent domain sizes (D-values) of Co_3_O_4_ and Co_2_Pd phases in the synthesized samples with respect to the corresponding crystallographic facets in CoO@Pd. (d) Relative percentage deviation of D_{hkl}_ values for Pd crystalline facets in the synthesized samples compared to those of the corresponding facets in CoO@Pd.

1. X-ray absorption spectroscopy analysis





**Figure S3** The enlarged post-edge region and H_P_/W_P_ ratios of (**a)** Co K-edge and (**b)** Cu K-edge XANES spectra of experimental samples CoO@Pd and Cu-AC.

1. Pd K-edge XANES analysis on CPCu catalysts





**Figure S4** Pd K-edge XANES spectra of Pd-AC, CoO@Pd and experimental samples.

**Table S2** Model determined structure parameters of CoO@Pd, CPCu-1 and CPCu-10 at Co, Pd and Cu K-edges.

| Edge | Sample | CN | | | | R (Å) | | | | Sigma square |
| --- | --- | --- | --- | --- | --- | --- | --- | --- | --- | --- |
|  |  | Cu-O | Cu-O2 | Cu-Pd | Cu-Cu | Cu-O | Cu-O2 | Cu-Pd | Cu-Cu |  |
| Cu | Cu-AC | 2.56 | 2.58 |  | 0.63 | 1.88 | 2.90 |  | 3.025 | 0.00465 |
|  | CPCu-1 | 3.56 |  | 1.07 | 0.88 | 1.88 |  | 2.576 | 2.948 |  |
|  | CPCu-2 | 3.09 |  |  |  | 1.95 |  |  |  |  |
|  | CPCu-10 | 2.69 | 1.51 | 0.51 | 0.88* | 1.91 | 3.28 | 2.848 | 2.501* |  |
| Edge | **Sample** | **CN** | | | | **R (Å)** | | | | **Sigma square** |
|  |  | Pd-O | Pd-Co | Pd-Pd | Pd-Cu | Pd-O | Pd-Co | Pd-Pd | Pd-Cu |  |
| Pd | Pd-AC | 0.86 |  | 8.01 |  | 2.12 |  | 2.747 |  | 0.00764 |
|  | CoO@Pd | 0.50 |  | 6.92 |  | 1.98 |  | 2.739 |  |  |
|  | CPCu-1 | 0.50 |  | 6.62 | 0.45 | 1.98 |  | 2.735 | 2.676 |  |
|  | CPCu-2 | 0.50 |  | 6.62 | 0.45 | 2 |  | 2.738 | 2.709 |  |
|  | CPCu-10 | 0.35 |  | 7.38 | 0.45 | 1.99 |  | 2.738 | 2.709 |  |
| Edge | **Sample** | **CN** | | | | **R (Å)** | | | | **Sigma square** |
|  |  | Co-O | Co-O2 | Co-Co |  | Co-O | Co-O2 | Co-Co |  |  |
| Co | Co-AC | 1.69 | 1.00 | 0.39** |  | 2.01 | 2.90 | 2.553** |  | 0.00389 |
|  | CoO@Pd | 3.54 |  |  |  | 2.90 |  |  |  |  |
|  | CPCu-1 | 2.7 | 2.13 | 1.57 |  | 2.06 | 3.01 | 3.119 |  |  |
|  | CPCu-2 | 2.65 | 1.74 | 1.51 |  | 2.06 | 3.02 | 3.118 |  |  |
|  | CPCu-10 | 2.59 | 2.70 | 1.15 |  | 2.06 | 3.03 | 3.136 |  |  |

　* **denote metallic bond pair of Cu-Cu ** denote metallic bond pair of Co-Co**

1. **XRD analysis on CPCu catalysts**





**Figure S5** Scherrer equation determined average coherent length of Pt-AC, Pd-AC, CoO@Pd and CPCu-1, CPCu-2 and CPCu-10.

1. Cu K-edge XANES analysis on CPCu catalysts





**Figure S6** Cu K-edge (a) XANES and (b) EXAFS of Cu_2_O, CuO, Cu-AC and CPCu-10.

1. Co K-edge XAS analysis on CPCu catalysts





**Figure S7** Co K-edge EXAFS spectra of Co-AC, CoO@Pd, CPCu catalysts and Co(OH)_2_.

1. EPR analysis on CoO@Pd, CPCu-1 and CPCu-10





**Figure S8** EPR spectra of CoO@Pd, CPCu-1 and CPCu-10.

1. WT-EXAFS analysis on CPCu catalysis





**Figure S9** WT-EXAFS spectra of M-AC (M denotes, Cu, Pd and Co), CoO@Pd and CPCu catalysis at Cu, Pd and Co K-edges.





**Figure S10** The peak width for scattering peaks of WT-EXAFS patterns for CPCu catalysts at (a) Co, (b) Pd and (c) Cu K-edges.





**Figure S11** WT-EXAFS patterns of (a) Co-AC, (b) Pd-AC and (c) Cu-AC at Co, Pd and Cu K-edge. respectively.





**Figure S12** PFY-XANES spectra of CoO@Pd, CPCu-1 and CPCu-10 at Pd, Co and Cu K-edges. (**20260406**)

1. PFY-XANES analysis of CoO@Pd, CPCu-1 and CPCu-10 under potential conditions of ORR





**Figure S13** The 1^st^ deviation curves of Co K-edge PFY-XANES spectra for CoO@Pd, CPCu-1 and CPCu-10 under potential driven conditions of ORR.





**Figure S14** Comparison of FT-EXAFS spectra of (a) CoO@Pd and (b) CPCu-1 at Co K-edge between as-prepared and Voc states.

1. Electrochemical analysis of CPCu catalysis





**Figure S15** (a) the CV curves (**Figure 6a**) and (b) it’s zoom-in from 0.2 to 0.9 V vs. RHE of samples in comparison.

In the context of recently reported Cu-based ORR catalysts, the present CPCu-1 system demonstrates a distinctly advanced performance envelope by simultaneously achieving record-level intrinsic activity, device-relevant power output, and exceptional durability. Prior Cu–N–C single-atom catalysts typically exhibit half-wave potentials in the range of 0.89–0.92 V vs. RHE, with kinetic current densities on the order of tens of mA cm⁻², while Cu-modified noble-metal catalysts rely on ligand and strain effects to enhance activity at the expense of precious metal content. In contrast, CPCu-1 adopts a tri-metallic division-of-labor strategy, in which Pd governs O₂ adsorption and initial activation, CoOₓ facilitates interfacial oxygen intermediate stabilization, and atomically dispersed CuOₓ serves as a highly efficient electronic regulator that optimizes the adsorption–desorption energetics of *OOH/*OH species.

As a result, CPCu-1 delivers an on-set potential as high as 0.935 V vs. RHE and a mass activity approaching 1.6 × 10⁴ mA mgCu⁻¹ at 0.85 V vs. RHE—exceeding commercial Pt/C by more than two orders of magnitude on a Cu-mass-normalized basis and outperforming most reported Cu-based ORR catalysts. Importantly, this superior intrinsic activity is directly translated into alkaline fuel cell performance, where CPCu-1 achieves a peak power density of approximately 430 mW cm⁻², nearly 60% higher than that of Pt/C, together with an open-circuit voltage of 0.935 V.

Beyond activity, CPCu-1 exhibits remarkable durability, maintaining stable ORR performance over 50,000 accelerated durability test (ADT) cycles without detectable degradation. Operando analysis reveals a characteristic three-stage durability evolution governed by dynamic self-reconstruction of the CuOₓ–CoOₓ interfacial domains, which continuously regenerates active sites and suppresses irreversible deactivation pathways. Collectively, these results position CPCu-1 among the most competitive Cu-involved ORR catalysts reported to date (see **Table S2**), establishing interfacial CuOₓ-mediated electronic regulation as a viable route toward high-efficiency and long-lifetime alkaline fuel cell cathodes.

**Figure S16** demonstrates the **(a)** Full potential sweeping range and enlarged potential ranging from 0.1 to 1.0 V vs. RHE in Figure 7(h).





**Figure S16 (a)** Full potential sweeping range and enlarged potential ranging from 0.1 to 1.0 V vs. RHE in Figure 6(h).

1. **Alkaline fuel cell module test conditions.**

Alkaline fuel cell (AFC) performance was evaluated in a single-cell membrane–electrode assembly (MEA) configuration with a geometric active area of 4 cm². The cathode catalyst consisted of CoOx-supported Pd nanoclusters with Cu decoration. Catalyst inks were prepared by dispersing the catalyst powder in a mixed solvent containing an anion-exchange ionomer, followed by ultrasonic homogenization. The ink was spray-coated onto a gas diffusion layer (GDL) to form the cathode catalyst layer. The total metal loading (Co + Pd + Cu) at the cathode was 8.0 mg per MEA, corresponding to 2.0 mg cm⁻². The anode consisted of a commercial Pt/C catalyst (J.M./Pt-C, 20 wt%) coated on a GDL. The MEA was assembled using an anion-exchange membrane (AEM, Fumasep FAA-3-50（Fumatech）), which was pre-treated in 1.0 M KOH for 24 h to convert the membrane into the OH⁻ form, followed by thorough rinsing with deionized water prior to cell assembly. The ionomer used in the catalyst layer was similarly pre-alkalized. Fuel-cell polarization and power-density curves were collected at 65 °C under fully humidified gas streams. High-purity hydrogen (H₂) and oxygen (O₂) were supplied to the anode and cathode, respectively, at 200 sccm, with a constant backpressure of 1 bar. The alkaline environment was maintained using 0.5–1.0 M KOH_(aq)_. Sufficient gas flow rates were ensured to eliminate mass-transport limitations under high current-density operation. Prior to data acquisition, the cell was conditioned at low current density until the open-circuit voltage (OCV) and internal resistance reached steady-state values. Polarization curves were recorded under a galvanostatic step mode, in which the current density was increased incrementally with adequate holding time at each step to ensure quasi-steady-state operation. The high-frequency resistance (HFR) was measured in situ by electrochemical impedance spectroscopy (EIS), and the reported cell voltages were corrected for ohmic losses (iR correction) unless otherwise specified.

**Table S3** The electrochemical results of J.M.-Pt/C, Co-AC, Pd-AC, Pd-1, Cu-AC PdCu (-1, -2, -10), PdCu (-1, -2, -10), CPCu (-1, -2, -10) and CPCu catalysts. Unit of Voc and E_1/2_ is V (V vs. RHE)

| Sample | V_oc_ | E_1/2_ | @ 0.85 V | | | @ 0.90 V | | |
| --- | --- | --- | --- | --- | --- | --- | --- | --- |
|  |  |  | J_K_  mAcm^-2^ | MA  (mAmg_Cu_^-1^) | MA  (mAmg_Pd+Cu_^-1^) | J_K_  mAcm^-2^ | MA  (mAmg_Cu_^-1^) | MA  (mAmg_Pd+Cu_^-1^) |
| J.M.-Pt/C | 0.910 | 0.844 | 4.37 | 67.0 | 67.0 | 1.21 | 24.9 | 24.9 |
| Co-AC* | 0.712 | 0.613 | 0.20 | 3.57 | N/A | N/A | N/A | N/A |
| Pd-AC* | 0.898 | 0.827 | 3.23 | N/A | 44.9 | 0.39 | N/A | 5.45 |
| Pd-1 | 0.835 | 0.781 | 0.31 | N/A | N/A | 0.03 | N/A | N/A |
| Cu-AC* | 0.720 | 0.666 | 0.01 | N/A | N/A | 0.01 | N/A | N/A |
| PdCu-1 | 0.844 | 0.798 | 0.45 | N/A | N/A | 0.04 | N/A | N/A |
| PdCu-2 | 0.849 | 0.812 | 0.85 | N/A | N/A | 0.10 | N/A | N/A |
| PdCu-10 | 0.891 | 0.847 | 4.47 | N/A | 56.48 | 0.49 | N/A | 6.22 |
| CoCu-1 | 0.726 | 0.674 | 0.01 | N/A | N/A | 0.01 | N/A | N/A |
| CoCu-2 | 0.726 | 0.665 | 0.01 | N/A | N/A | 0.01 | N/A | N/A |
| CoCu-10 | 0.726 | 0.664 | 0.01 | N/A | N/A | 0.01 | N/A | N/A |
| CoO@Pd | 0.899 | 0.857 | 5.37 | N/A | 108.9 | 0.66 | N/A | 13.38 |
| CPCu-1 | 0.935 | 0.876 | 17.47 | 16476.7 | 242.3 | 2.09 | 1975.5 | 29.1 |
| CPCu-2 | 0.961 | 0.911 | 35.36 | 16746.7 | 485.5 | 10.29 | 4874.4 | 141.3 |
| CPCu-10 | 0.970 | 0.920 | 59.78 | 4923.9 | 748.1 | 15.60 | 1285.1 | 195.2 |

* for Co-AC, Cu-AC and Pd-AC the MA is calculated by normalizing with the loading of Co, Cu and Pd atoms.

* for PdCu-1 and CoCu-1 the Cu loading is 1 wt%.

**Table S4** Benchmark for the ORR performance of CPCu-1 and Cu based samples in ORR and AFC

| Catalyst type | Sample name | ORR performance | | | Fuel-cell / Device performance | Key factors | Ref. |
| --- | --- | --- | --- | --- | --- | --- | --- |
|  |  | MA / SA | Onset / E1/2 (V vs RHE) | ADT durability |  |  |  |
| Atomic CuOx clusters | CPCu-1 | 16,476 mA mg_Cu_^-1^  @0.85 V  1,975 mA mg_Cu_^-1^ @0.90 V | 0.935  **E_1/2_ = 0.876** | 50000 | AFC ~430 mW cm⁻² | Atomic CuO_x_-Pd-CoO_x_ active site | This work |
| **Cu single-atom / Cu-N-C** | **Cu₁/NC-900** | Jk = **35.1 mA cm⁻² @0.85 V** | **E_1/2_ = 0.894** | ΔE1/2 < 5 mV / 10k | Zn–air > Pt/C | Cu-N₃ + defect active site | Nano Res. 2021, 14(4): 998–1003 |
|  | **Cu-TCNQ/GO (pyrolysis-free)** | — | **E_1/2_ = 0.92** | Stable | **APEFC 320 mW cm⁻²** | Tunable atomic Cu-Nx structure | Chinese Chemical Letters 2025, 36 109630 |
|  | **CuSA-NP@NC** | — | 0.870 (↑ from 0.842) | 86.6% @ 24 h | Zn–air **224 mW cm⁻²** | Cu NP modulate electronic structure of SA | Next Materials 2024, 3, 100162 |
| **Cu-modified noble metal** | **PtCuNC-700** | **0.45 A mgPt⁻¹ @0.90 V** | **E_1/2_ = 0.92** (acid) | ΔE ≈ 17 mV / 30k | **PEMFC 930 mW cm⁻²** | Cu induced ligand + strain stabilization | CCS Chem. 2023, 5, 2545–2556 |
|  | **PtCuAu porous film** | 0.871 A mgPt⁻¹ | ~0.88 | Best in set | — | Cu stabilized the electronic structure of alloy | ACS Catal. 2020, 10, 9967−9976 |
| **Non-PGM AFC systems** | **Ni@CNx // CoMn spinel** | — | — | >100 h stable | **APEFC ~200–210 mW cm⁻²** | Milestone of none noble metal AFC | PNAS 2022, 119, 13, e2119883119 |
| **Spinel oxides (Cu-relevant context)** | **CoMn₂O₄/C** | — | **E_1/2_ = 0.89** | Stable | AFC-relevant | Cu/Co/Mn charge transfer | PNAS 2019, 116, 49, 24425–24432 |
| **Durability concept** | **SiO₂/Fe-N-C SAC** | — | ~0.90 | **ΔE_1/2_ = 5 mV / 30k** | — | ROS/H₂O₂ suppression mechanism | Nat. Commun. 2025, 16, 10178 |
